# Electrostatic tuning of Na_V_ channel activation by the domain II S3–S4 extracellular loop

**DOI:** 10.1113/JP291286

**Published:** 2026-07-25

**Authors:** Ashvriya Thapa, Lotten Ragnarsson, Hue Tran, Angelo Keramidas, Jennifer R. Deuis, Irina Vetter

**Affiliations:** ^1^ Institute for Molecular Bioscience The University of Queensland St Lucia Queensland Australia; ^2^ School of Pharmacy The University of Queensland Woolloongabba Queensland Australia

**Keywords:** extracellular surface charge, gating‐modifier peptides, Na_V_1.7, voltage‐gated sodium channels, voltage‐sensor activation

## Abstract

**Abstract:**

How Na_V_ channel isoforms set their activation voltages – and thereby define their specific functional roles in excitable cells – remains poorly understood. We show that extracellular charges in the domain II (DII) S3–S4 loop critically tune voltage‐sensor activation in Na_V_1.7. Introducing positively charged residues in this loop depolarizes activation, whereas additional negative charges shift it in the hyperpolarizing direction. Extracellular Ca^2^^+^ further modulates these effects through electrostatic screening, demonstrating that local surface charge is a key determinant of voltage‐sensor function. The Na_V_1.7‐selective spider toxin Pn3a, which binds the DII S3–S4 loop, produces enhanced depolarizing shifts when its net positive charge is increased, showing that ligand electrostatics can fine‐tune gating. Together, these results demonstrate that the local electrostatic environment near the DII voltage sensor controls Na_V_1.7 activation, providing a general mechanism – likely conserved across all voltage‐gated ion channels – by which loop charges, extracellular ions or bound ligands can modulate channel function. By showing that local electrostatics near the DII voltage sensor govern activation, our findings provide a mechanistic explanation for isoform‐specific gating and a framework for precisely tuning Na_V_ channel function with engineered charged ligands.

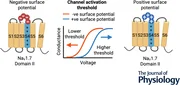

**Key points:**

- Voltage‐gated sodium (Na_V_) channels are essential for generating electrical signals in nerves, muscles and the heart, yet how different Na_V_ types are tuned to open at specific voltages was not well understood.
- This study shows that charged amino acids on the outer part of Na_V_1.7 help set the voltage at which the channel activates, with positive charges making it harder to open and negative charges making it easier.
- We find that extracellular calcium can influence this process, demonstrating that the local electrical environment around the channel shapes its activity.
- A naturally occurring spider toxin, Pn3a, can also adjust channel activation depending on its surface charge, suggesting that we can engineer ligands to fine‐tune excitability.
- Together, these results explain how Na_V_ channels achieve isoform‐specific gating and provide a framework for designing molecules that selectively control activation, with potential implications for pain and other disorders.

## Graphical abstract

**Abstract figure legend** Voltage‐gated sodium (Na_V_) channels are critical for electrical signalling, yet the molecular determinants that tune isoform‐specific activation remain incompletely understood. Here, we show that charged amino acids in the extracellular DII S3–S4 loop of Na_V_1.7 play a key role in setting voltage‐dependent activation. Positively charged residues in this region shift channel opening to more depolarized potentials, raising the threshold for activation, whereas negatively charged substitutions facilitate activation at more hyperpolarized voltages, lowering the threshold for activation. This is consistent with electrostatic modulation of voltage‐sensor movement and altered interactions between the voltage‐sensing domain and the local extracellular surface potential. Together, these findings define a mechanism by which extracellular charge elements in the DII S3–S4 loop regulate Na_V_1.7 gating, providing a framework for understanding how surface electrostatics contribute to isoform‐specific sodium channel activation and excitability.

## Introduction

Voltage‐gated sodium (Na_V_) channels are essential pore‐forming membrane proteins that open upon membrane depolarization to allow rapid flux of Na^+^ across the membrane. This Na^+^ influx drives the rising phase of the action potential, sets the threshold for neuronal firing, and determines the excitability of muscle, cardiac and neuronal cells. Dysfunction or aberrant modulation of Na_V_ channels is associated with a wide range of pathophysiological conditions, including epilepsy, cardiac arrhythmias, neuropathic pain and muscle disorders, making them important therapeutic targets (Ahern et al., 2016).

Na_V_ channels contain a single α‐subunit organized into four homologous domains (DI–DIV), each containing six transmembrane α‐helices (S1–S6). Within each domain, the S1–S4 helices form a voltage sensing domain (VSD), whereas the S5 and S6 helices from all four domains assemble to form the ion conducting pore. The S4 helix contains regularly spaced positively charged residues, typically arginine or lysine, positioned at nearly every third residue along the helix, which act as the primary gating charges that move outward upon membrane depolarization to open the pore (Payandeh et al., 2011). Each of the four different S4 voltage sensors in DI–DIV exhibits functional specialization, with DI to DIII mediating rapid channel activation, whereas DIV couples activation to fast inactivation (Brake et al., 2022; Capes et al., 2013; Chanda & Bezanilla, 2002; Goldschen‐Ohm et al., 2013). This asynchronous activation reflects the fact that, although homologous, the four Na_V_ domains are not identical and exhibit distinct structural and energetic specializations.

The effective voltage sensed by the S4 gating charges is influenced not only by the transmembrane potential, but also by the local potential created by fixed charges on the channel protein and surrounding membrane. Negatively charged amino acid residues and anionic membrane phospholipids dominate the net surface charge density and generate a surface potential that biases voltage‐sensor activation toward more hyperpolarized membrane potentials (Cukierman et al., 1988; Hille et al., 1975). This surface potential can be attenuated by protonation (e.g., at lower extracellular pH), by divalent cations bound to acidic groups, or by freely diffusible divalent ions that screen the local electric field, shifting the voltage dependence of activation toward more depolarized potentials (Hille, 1968; Hille et al., 1975). In addition, ligand interactions can alter the local electrostatic environment near the S4 segment and affect channel voltage sensitivity (Thapa et al., 2024).

Nine mammalian Na_V_ isoforms (Na_V_1.1–Na_V_1.9) have been identified, each exhibiting distinct tissue expression, gating properties, and drug and toxin sensitivities. Among these, Na_V_1.7 and Na_V_1.8 are prominently expressed in peripheral sensory neurons and play complementary roles in nociception (Rush et al., 2007). Na_V_1.7 activates at relatively hyperpolarized membrane potentials, with a midpoint of activation (*V*_50_) ranging from –30 to –20 mV (Deuis et al., 2021; Köster et al., 2025; Vijayaragavan et al., 2001), enabling it to act as a threshold‐setting channel that amplifies small depolarizations. In contrast, Na_V_1.8 activates at more depolarized potentials, with a *V*_50_ ranging from –5 to +5 mV (Deuis et al., 2021; Köster et al., 2025; Vijayaragavan et al., 2001), and exhibits slower gating kinetics and reduced steady‐state inactivation, allowing it to sustain repetitive firing once threshold has been reached. Despite extensive conservation of the voltage sensing helices, the mechanisms that tune activation thresholds between Na_V_ isoforms, and the role of extracellular regions of the channel in this process, remain poorly understood.

The transmembrane helices of Na_V_ channels are connected by extracellular loops that provide critical interaction surfaces for gating‐modifier toxins. These toxins bind to the channel loops near the S4 helix (often at the S3–S4 linkers), stabilizing resting or activated voltage sensor conformations, and thereby shift channel activation or inactivation without occluding the pore. Toxins that preferentially associate with the voltage‐sensing domains of DII typically shift channel activation, whereas those targeting DIV more often perturb fast inactivation, consistent with the specialized functional roles of these domains in gating (Bosmans et al., 2008; Deuis, Mueller et al., 2017). These observations suggest that extracellular loops are ideally positioned to influence voltage sensor movement, yet their intrinsic contribution to Na_V_ channel activation in the absence of toxins has not been systematically investigated.

In this study, we show that extracellular surface charges on Na_V_ channels, and in particular the net charge of the DII S3–S4 loop, play a central role in tuning voltage‐sensor activation. Positive charges in this loop shift activation to more depolarized potentials, whereas negative charges shift it to more hyperpolarized potentials. Extracellular divalent cations, such as Ca^2^^+^, modulate these effects by screening surface charges, and increasing the positive surface charge of ligands, such as the Na_V_1.7‐selective spider toxin Pn3a, which binds the DII S3–S4 loop, enhances their inhibitory activity. Together, these findings demonstrate that modulation of the surface charge near DII – whether by intrinsic extracellular loop charges, mobile ions, or bound ligands – shapes the local electrostatic environment around the S4 voltage sensor to control Na_V_ channel activation.

## Methods

### Cell culture

Human Embryonic Kidney (HEK) 293 cells stably expressing the human Na_V_ β‐subunits β1/β2 (SB Drug Discovery, Glasgow, UK) were cultured in minimum essential medium (MEM) supplemented with 10% fetal bovine serum (FBS) and 2 mM l‐glutamine. ND7/23 cells (The European Collection of Cell Cultures, Porton Down, UK) were cultured in Dulbecco's modified Eagle's medium (DMEM) supplemented with 10% FBS. Cells were grown in an incubator at 37°C with 5% CO_2_ and passaged every 3–4 days (at 90% confluency).

### Mutagenesis

Wild‐type (WT) human Na_V_1.7 cDNA (NM_002977; kindly provided by Dr James Cox, University College London) and WT human Na_V_1.8 cDNA (NM_006514.3; GenScript Corporation, Singapore), cloned into the pcDNA3.1 mammalian expression vector, were subjected to *in vitro* site‐directed mutagenesis using the QuikChange™ II XL Site‐Directed Mutagenesis Kit (Agilent Technologies, Santa Clara, CA, USA). Sequences for all extracellular loop swaps and charge substitutions are provided in Tables 1 and 2. The mutations were confirmed by Sanger sequencing at the Brisbane node of the Australian Genome Research Facility.

**Table 1:** Sequences for Na_V_1.7 extracellular loop replacement mutants

Loop	Na_V_1.7	Sequence
DI S3–S4	WT	AYLT EFVNLGNVSALR
	KKKK	AYLTEFV **KKKK** VSALR
DII S1–S2	WT	LFMAM **E** HHPMT **EE** FKN
	Na_V_1.8	LFMAM **E** HHGMSPTFKN
DII S3–S4	WT	SLV **E** LFLA **D** V **E** GLSV
	Na_V_1.8	SLV **E** LGVA **KK** GSLSV
	KKKK	SLV **E** LGVA **KKKK** LSV
	EEEE	SLV **E** LGVA **EEEE** LSV
DIII S3–S4	WT	ANTLGYSDLGPIKS
	KKKK	ANTLGYS **KKKK** IKS
DIV S1–S2	WT	TMMV **E** K **E** GQSQHMT
	Na_V_1.8	TMMV **E** TDDQS **E** HMT
DIV S3–S4	WT	IET‐ ‐ ‐YFVSPTL
	Na_V_1.8	IETLQSYF‐SPTL
	KKKK	IET‐ ‐ ‐ **KKKK** PTL

Grey highlights the transmembrane region.

**Table 2:** Sequences for Na_V_1.8 extracellular loop replacement mutants

Loop	Na_V_1.8	Sequence
DII S3–S4	WT	SLL **E** LGVA **KK** GSLSV
	Na_V_1.7	SLL **E** LFLA **D** V **E** GLSV
	EEEE	SLLELGVA **EEEE** LSV

Grey highlights the transmembrane region.

### Transfection

Na_V_1.7 channels were transiently transfected into HEK293 cells stably expressing β1/β2 subunits using Lipofectamine 2000 (Thermo Fisher Scientific, Scoresby, VIC, Australia) as per the manufacturer's instructions. Na_V_1.8 channels were transiently transfected into ND7/23 cells using the calcium phosphate method. After 24 h at 37°C, the medium containing transfection reagents was replaced, and cells were incubated at 28°C for 24 h prior to patch‐clamp experiments.

### Automated patch‐clamp electrophysiology

Automated whole‐cell patch‐clamp recordings were performed with a QPatch‐16 automated electrophysiology platform (Sophion Bioscience, Ballerup, Denmark) using single‐hole (QPlate 16 with a standard resistance of 2 ± 0.4 MΩ) on HEK293 cells transiently expressing WT or mutant Na_V_1.7. Whole‐cell currents were filtered at 8 kHz and acquired at 25 kHz and the linear leak was corrected by *P*/4 subtraction (leak potential −90 mV, leak sweep amplitude 10%). Series resistance across recorded cells ranged between 5 and 10 MΩ and was compensated at 70%. To minimize series resistance errors, only cells with currents that ranged between 1 and 5 nA were selected.

The extracellular solution (ECS) consisted of (in mM): 70 NaCl, 70 choline chloride, 4 KCl, 2 CaCl_2_, 1 MgCl_2_, 10 HEPES and 10 glucose, pH to 7.4 with NaOH (adjusted to 305 mOsm/l with sucrose). The intracellular solution (ICS) consisted of (in mM): 140 CsF, 1 EGTA, 5 CsOH, 10 HEPES and 10 NaCl, pH to 7.3 with CsOH (adjusted to 320 mOsm/L with sucrose).

Current–voltage (*I–V*) curves were obtained with a holding potential of −90 mV followed by a series of 50 ms step pulses that ranged from −110 to +80 mV in 5 mV increments (repetition interval 6 s). Conductance–voltage (*G*–*V*) plots for peak current were obtained by calculating the conductance (*G*) at each voltage (*V*) using the equation *G*  =  *I*/(*V* − *V*_rev_), where *V*_rev_ is the reversal potential. *G*–*V* curves were fitted with a Boltzmann equation. From this equation the *V*_50_, defined as the membrane voltage at which 50% of channels are open, was computed. The voltage dependence of steady‐state fast inactivation was examined using a 10 ms pulse of −20 mV immediately after a series of 500 ms step pulses that ranged from −110 to +80 mV in 5 mV increments (repetition interval 6 s) to assess the available non‐inactivated channels. The peak current at each test pulse was normalized and fitted using a Boltzmann equation.

Concentration–response curves at Na_V_1.7 were acquired using a holding potential of −90 mV and a 50 ms pulse to −20 mV every 20 s (0.05 Hz). Pn3a and its charge enhanced mutant were diluted in ECS with 0.1% bovine serum albumin (BSA) and peptides were incubated with cells for 5 min before measurements were made. Peak current was normalized to buffer control and fitted to a four‐parameter Hill equation with variable Hill coefficient.

### Conventional patch‐clamp electrophysiology

Conventional whole‐cell patch‐clamp recordings were performed on ND7/23 cells transiently expressing WT or mutant hNa_V_1.8 seeded onto 12 mm poly‐d‐lysine‐coated coverslips. Whole cell experiments were recorded using an Axon 700B amplifier (Molecular Devices, Can Jose, CA, USA). Patch electrodes were made from borosilicate glass capillaries (G150F‐3; Warner Instruments, Hamden, CT, USA) and heat‐polished to a final resistance of 2–4 MΩ when filled with intracellular solution. Currents were filtered at 2 kHz and sampled at 50 kHz and the linear leak was corrected by *P*/4 subtraction. Whole‐cell data were accepted for analysis if the series resistance of the recorded cell was ≤12 MΩ and was compensated for by ≥60%.

The ECS consisted of (in mM): 140 NaCl, 5 KCl, 1 MgCl_2_, 2 CaCl_2_, 10 HEPES and 10 glucose adjusted to pH 7.4 with NaOH. Tetrodotoxin (TTX; 1 µM) was added to the ECS to block endogenous Na_V_ channels. The ICS consisted of (in mM): 10 NaCl, 10 HEPES, 5 EGTA and 140 CsCl adjusted to pH 7.4 with CsOH.

*I–V* curves were obtained with a holding potential of −80 mV followed by a series of 50 ms step pulses that ranged from −80 to +80 mV in 5 mV increments (repetition interval 6 s). *G*–*V* plots for peak current were fitted with a Boltzmann equation as described above. The voltage dependence of steady‐state fast inactivation was examined using a 10 ms pulse of +10 mV immediately after a series of 500 ms step pulses that ranged from −80 to +80 mV in 5 mV increments (repetition interval 6 s) to assess the available non‐inactivated channels and fitted using a Boltzmann equation as described above.

For calcium screening experiments, conventional whole‐cell patch‐clamp recordings were performed on HEK293 cells stably expressing β1/β2 and transiently expressing WT or mutant hNa_V_1.7 seeded onto 12 mm poly‐d‐lysine‐coated coverslips. The ECS consisted of (in mM): 145 NaCl, 0–50 CaCl_2_ (as stated), 10 mM HEPES and 10 mM glucose adjusted to pH 7.4 with NaOH. The ICS consisted of (in mM): 145 NaCl, 10 HEPES and 5 EGTA, adjusted to pH 7.4 with NaOH. *I–V* curves were obtained with a holding potential of −110 mV followed by a series of 50 ms step pulses that ranged from −110 to +80 mV in 5 mV increments (repetition interval 6 s). *G*–*V* plots for peak current were fitted with a Boltzmann equation as described above.

### Peptide synthesis

Pn3a and Pn3a[D1K,D8K,E10K,D12K] were assembled on a Liberty Prime synthesizer (CEM Corporation, Matthews, NC, USA) using pre‐loaded Fmoc‐Lys Wang resin as described previously (Deuis, Dekan et al., 2017). Peptides were cleaved from the resin and simultaneously deprotected and then precipitated with ice‐cold diethyl ether. Peptides were purified using analytical HPLC on a Shimadzu LC‐20AT system and mass determined using a SCIEX (Marlborough, MA, USA) API 2000 LC/MS/MS high performance triple quadrupole mass spectrometer equipped with an Agilent–Hewlett‐Packard 1100 series HPLC system. Linear Pn3a and Pn3a analogues were folded in a salt buffer containing 4.5 M NH_4_OAc/2 M urea/100 eq GSH/10 eq GSSG at a concentration of 0.1 mg/mL after 48 h at 4°C.

### Electrostatic surface potential calculations

Electrostatic surface potentials of the peptide Pn3a were calculated using the Adaptive Poisson–Boltzmann Solver (APBS) software package using the NMR structure (PDB ID: 5T4R) (Jurrus et al., 2018). Electrostatic potential maps were generated and visualized using PyMOL, with surface potentials displayed on the solvent‐accessible surface of Pn3a and contoured between −5 and +5 *kT*/*e*.

### Data analysis

Data were plotted and analysed using GraphPad Prism version 10.4.1 (GraphPad Software, Boston, MA, USA). Statistical significance was defined as *P* < 0.05 using tests as indicated. Data are presented as means ± standard deviation (SD) with the number of cells (*n*) provided in the figure legends and tables.

## Results

### Substitution of Na_V_1.8 DII S1–S2 and S3–S4 extracellular loops into Na_V_1.7 shifts activation to more depolarized potentials

**Figure 1:**
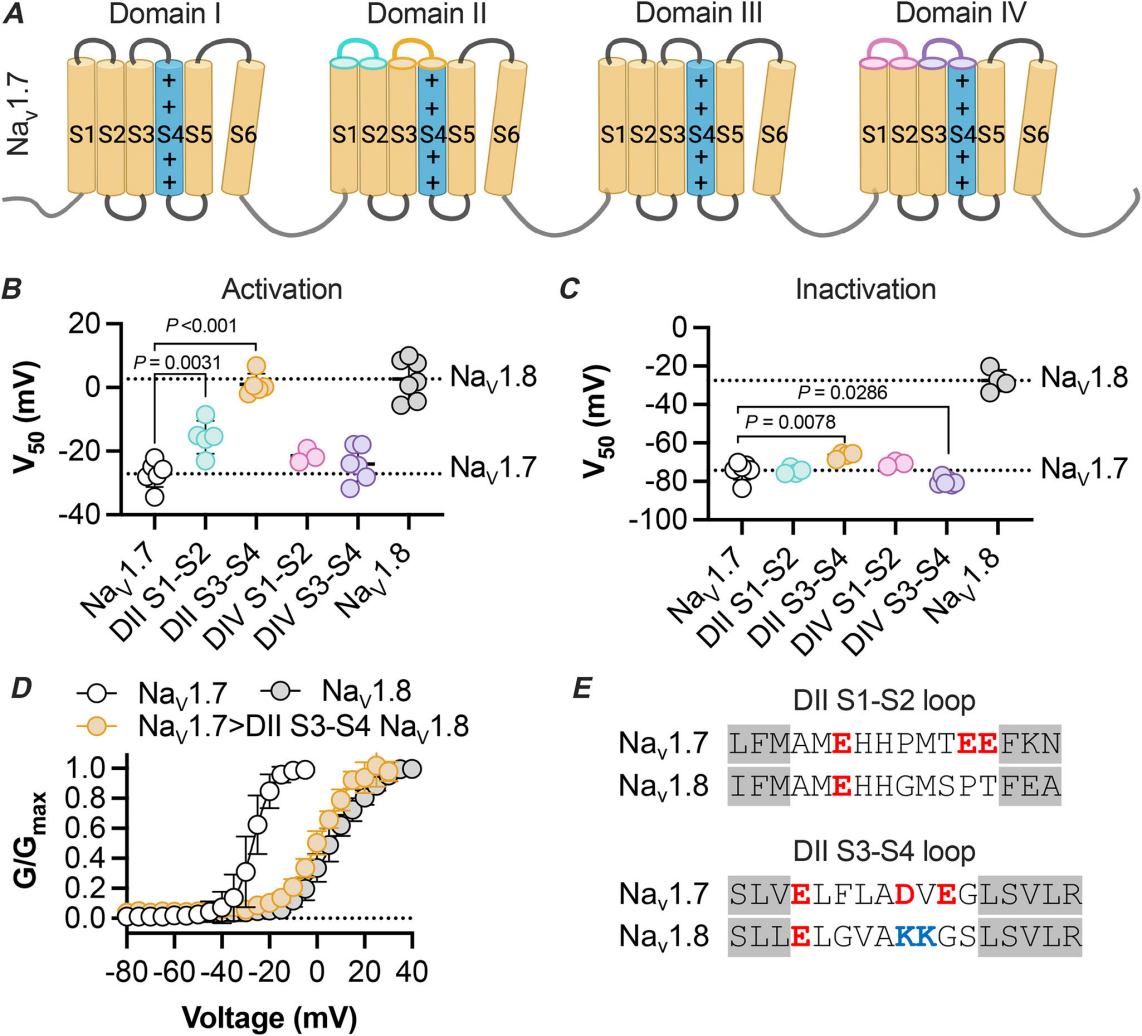
Extracellular loop chimeras identify the DII S3–S4 loop as a key determinant of Na_V_1.7 activation *A*, schematic illustration of Na_V_1.7 showing the DII and DIV extracellular loops that were replaced with the corresponding loops from Na_V_1.8. *B* and *C*, voltage dependence of activation (*B*) and inactivation (*C*) for wild‐type (WT) Na_V_1.7, WT Na_V_1.8 and Na_V_1.7 chimeras in which individual DII or DIV extracellular loops (S1–S2 or S3–S4) were replaced with the corresponding Na_V_1.8 sequences (*n* = 3–7 cells). Statistical significance was determined using one‐way ANOVA with Dunnett's multiple comparisons test compared to wild‐type Na_V_1.7. *D*, conductance–voltage (*G–V*) relationship of WT Na_V_1.7, WT Na_V_1.8 and Na_V_1.7 chimera with DII S3–S4 replacement to Na_V_1.8 (*n* = 4–6 cells). *E*, sequence comparison of the DII S1–S2 and S3–S4 loops of Na_V_1.7 and Na_V_1.8. Grey shading indicates transmembrane regions, while negatively and positively charged residues are indicated by red and blue, respectively.

Na_V_1.8 activates at substantially more depolarized potentials than TTX‐sensitive channels such as Na_V_1.7 (Deuis et al., 2021; Köster et al., 2025; Vijayaragavan et al., 2001), but the structural determinants underlying this difference remain unclear. Extracellular loops connecting S1–S2 and S3–S4 are critical neurotoxin‐binding sites, with interactions at DII influencing activation and those at DIV influencing inactivation (Bosmans et al., 2008; Deuis, Mueller et al., 2017). To investigate whether these extracellular loops contribute to voltage sensitivity, we generated chimeric Na_V_1.7/Na_V_1.8 channels in which the DII and DIV S1–S2 and S3–S4 extracellular loops of Na_V_1.7 were replaced with the corresponding loops from Na_V_1.8 (Fig. 1*A*).

Wild‐type (WT) Na_V_1.7 activated at a half‐maximum voltage (*V*_50_) of –27 ± 4 mV, whereas WT Na_V_1.8 exhibited a significantly depolarized *V*_50_ of 3 ± 6 mV, consistent with literature values (Fig. 1*B*; Deuis et al., 2021; Köster et al., 2025; Vijayaragavan et al., 2001). Replacing the DII S1–S2 or S3–S4 extracellular loops of Na_V_1.7, separately, with the corresponding loops from Na_V_1.8 produced significant shifts in activation. By contrast, the DIV loop substitutions had minimal effects on the *V*_50_ of inactivation (Fig. 1*C*; Table 3). DII S1–S2 replacement shifted the *V*_50_ of activation to –16 ± 5 mV, whereas DII S3–S4 replacement shifted the *V*_50_ to 1 ± 3 mV, closely resembling that of Na_V_1.8 and implicating this loop in isoform‐specific voltage sensitivity (Fig. 1*D*).

**Table 3:** Voltage‐dependence of activation and inactivation of Na_V_1.7/Na_V_1.8 loop mutants

Channel constructs	*V*_50_ of activation (mV)	*P*	*n* (cells)	*V*_50_ of inactivation (mV)	*P*	*n* (cells)
Na_V_1.7 WT	−27 ± 4	—	7	−74 ± 5	—	6
Na_V_1.7/1.8 DII S1–S2	−16 ± 5^*^	0.0031	5	−75 ± 2	>0.999	4
Na_V_1.7/1.8 DII S3–S4	1 ± 3^*^	<0.001	5	−66 ± 1^*^	0.0078	4
Na_V_1.7/1.8 DIV S1–S2	−22 ± 2	0.376	3	−71 ± 1	0.505	3
Na_V_1.7/1.8 DIV S3–S4	−24 ± 5	0.722	6	−80 ± 2^*^	0.0286	6
Na_V_1.8 WT	3 ± 6^*^	<0.001	7	−28 ± 6^*^	<0.001	4

Data represented as means ± SD. Activation and inactivation *V*_50_ of channel mutants compared to Na_V_1.7 WT using an ordinary one‐way ANOVA with Dunnett's post test.

* *P*<0.05.

Sequence analysis of the DII extracellular loops revealed pronounced charge differences between isoforms. Whereas the S1–S2 loop of Na_V_1.8 lacks two negatively charged glutamates present in Na_V_1.7, the S3–S4 loop carries two positively charged lysines *versus* two negative residues in Na_V_1.7 (Fig. 1*E*). This strong net positive charge in the S3–S4 loop of Na_V_1.8 correlates with the largest depolarizing (positive) shift in activation, prompting us to examine how charges in the loops influence voltage‐dependent gating.

### The charged residues in the DII S3–S4 extracellular loop are key determinants of Na_V_ activation threshold

**Figure 2:**
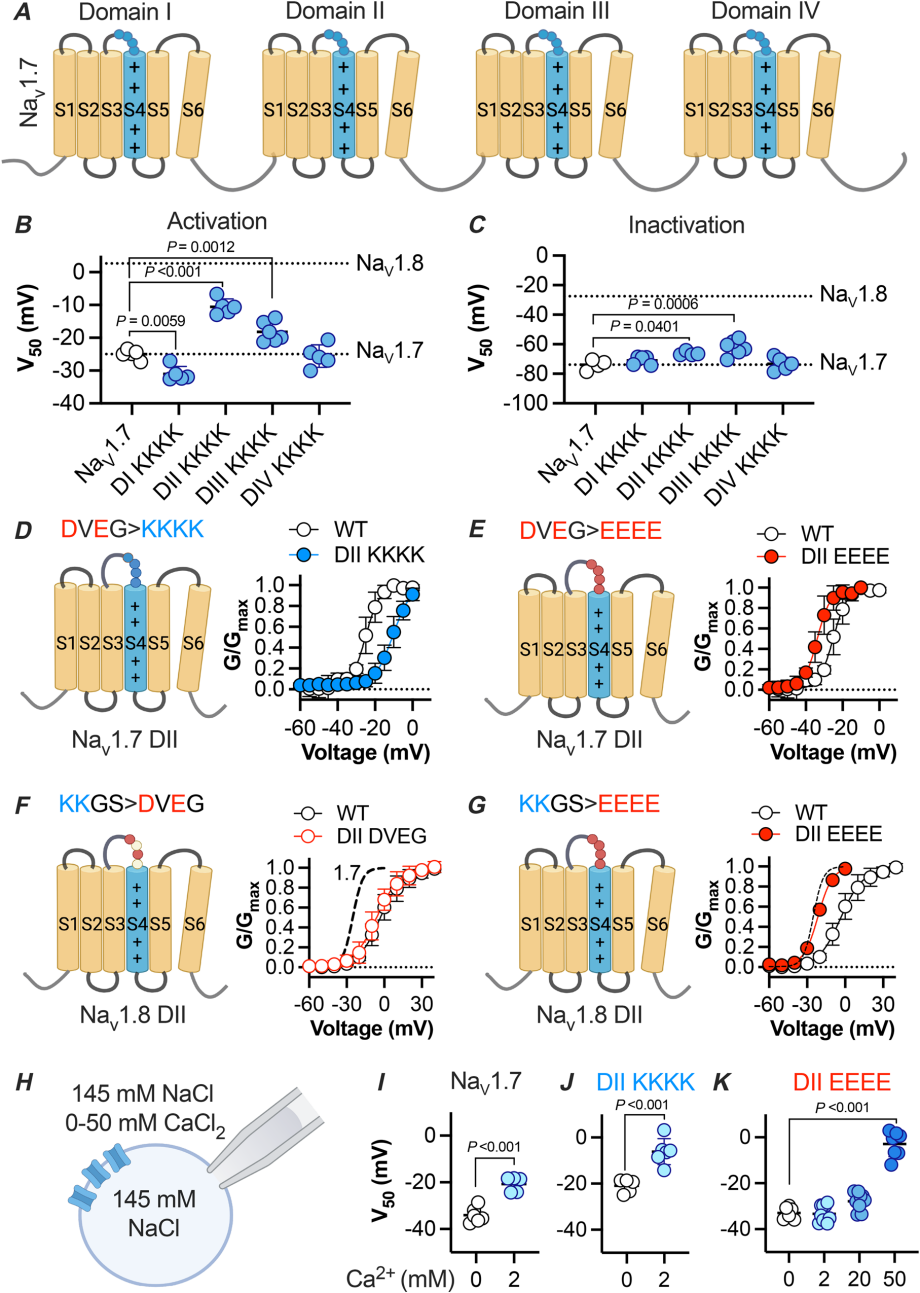
Electrostatic charge in the DII S3–S4 extracellular loop regulates Na_V_1.7 and Na_V_1.8 activation *A*, a contiguous stretch of four positively charged lysine residues (KKKK) was introduced into the extracellular S3–S4 loop immediately adjacent to the S4 segment in each domain (DI–DIV) of Na_V_1.7. *B* and *C*, voltage dependence of activation (*B*) and inactivation (*C*) for WT Na_V_1.7 and Na_V_1.7 DI–DIV S3–S4 loop KKKK mutants (*n* = 5–6 cells). Statistical significance was determined using one‐way ANOVA with Dunnett's multiple comparisons test compared to wild‐type Na_V_1.7. *D* and *E*, conductance–voltage (*G–V*) relationship of WT Na_V_1.7 (DVEG) compared to Na_V_1.7 DII positively charged mutant (KKKK) (*D*) and Na_V_1.7 DII negatively charged mutant (EEEE) (*E*) (*n* = 5–6 cells). *F* and *G*, *G–V* relationship of WT Na_V_1.8 (KKGS) compared to Na_V_1.8 chimera with DII S3–S4 replacement by Na_V_1.7 (DVEG) (*F*) and Na_V_1.8 DII negatively charged mutant (EEEE) (*G*) (*n* = 5–10 cells). *H*, schematic diagram representing the recording solutions used for Ca^2+^ screening experiments. *I–K*, voltage dependence of activation for WT Na_V_1.7 (DVEG) (*I*), Na_V_1.7 DII positively charged mutant (KKKK) (*J*), and Na_V_1.7 DII negatively charged mutant (EEEE) (*K*), with varying levels of extracellular Ca^2+^ (*n* = 5–10 cells). Statistical significance was determined using unpaired *t* test or one‐way ANOVA with Dunnett's multiple comparisons test compared to 0 mM Ca^2+^.

To directly test the effect of positive surface charge adjacent to the S4 voltage sensors, we introduced a contiguous stretch of four positively charged lysines (KKKK) into the extracellular S3–S4 loop closest to S4 in each domain (DI–DIV) of Na_V_1.7, separately, as described previously for K_V_ channels (Swapna et al., 2018), and assessed changes in channel activation (Fig. 2*A*).

Insertion of the KKKK motif produced domain‐specific effects on activation. Domain II showed the largest depolarizing shift, with *V*_50_ changing from –25 ± 1 mV in WT to –11 ± 2 mV, followed by Domain III (*V*_50_ –18 ± 3 mV). By contrast, Domain I exhibited a hyperpolarizing shift (*V*_50_ –31 ± 2 mV), whereas Domain IV showed no significant change (*V*_50_ –26 ± 3 mV) compared with WT (Fig. 2*B*, Table 4). Domain II showed the largest depolarizing shift, reinforcing its dominant role in activation (Fig. 2*D*). Comparatively small depolarizing shifts in the voltage dependence of inactivation were also observed for the DII and DIII KKKK mutants (Fig. 2*C*, Table 4).

**Table 4:** Voltage‐dependence of activation and inactivation of Na_V_1.7 KKKK and EEEE loop mutants

Channel Constructs	*V*_50_ of activation (mV)	*P*	*n* (cells)	*V*_50_ of Inactivation (mV)	*P*	*n* (cells)
Na_V_1.7 WT	−25 ± 1	—	5	−74 ± 3	—	4
Na_V_1.7 DI KKKK	−31 ± 2^*^	0.0059	5	−71 ± 3	0.620	5
Na_V_1.7 DII KKKK	−11 ± 2^*^	<0.001	5	−66 ± 1^*^	0.0401	4
Na_V_1.7 DIII KKKK	−18 ± 3^*^	0.0012	6	−62 ± 5^*^	0.0006	6
Na_V_1.7 DIV KKKK	−26 ± 3	0.990	5	−73 ± 4	0.997	5
Na_V_1.7 DII EEEE	−33 ± 3^*^	<0.001	6	−76 ± 2	0.7821	5

Data represented as means ± SD. Activation and inactivation *V*_50_ of channel mutants compared to Na_V_1.7 WT using an ordinary one‐way ANOVA with Dunnett's post test.

* *P*<0.05.

To test whether additional negative charges would shift activation in the opposite direction, we replaced the existing DII S3–S4 sequence (DVEG) in Na_V_1.7 with a highly negative EEEE motif. This substitution caused a pronounced hyperpolarizing shift in activation (*V*_50_ –33 ± 3 mV), supporting the notion that net extracellular loop charge is a key determinant of voltage sensitivity (Fig. 2*E*).

We next assessed whether altering the net surface charge of DII S3–S4 in Na_V_1.8 could produce comparable effects. Replacing the native KKGS motif with the Na_V_1.7 DVEG loop, which removes two positive charges and introduces two negative residues, did not significantly change the voltage dependence of activation (*V*_50_ –4 ± 5 mV *vs*. WT Na_V_1.8 –2 ± 5 mV; Fig. 2*F*, Table 5). In contrast, introducing the highly negative EEEE motif produced a pronounced hyperpolarizing shift in activation (*V*_50_ –24 ± 4 mV), similar to Na_V_1.7 (Fig. 2*G*, Table 5).

**Table 5:** Voltage‐dependence of activation and inactivation of Na_V_1.8 DII loop mutants

Channel constructs	*V*_50_ of activation (mV)	*P*	*n* (cells)
Na_V_1.8 WT	−2 ± 5	—	8
Na_V_1.8 DII DVEG	−4 ± 5	0.754	10
Na_V_1.8 DII EEEE	−24 ± 4^*^	<0.001	5

Data represented as means ± SD. Activation *V*_50_ of channel mutants compared to Na_V_1.8 WT using an ordinary one‐way ANOVA with Dunnett's post test.

* *P*<0.05.

### Extracellular Ca^2^^+^ modulates Na_V_1.7 activation by screening surface charges

Given that surface charges influence voltage sensitivity, we next tested whether extracellular divalent cations could modulate activation through electrostatic screening. We tested WT Na_V_1.7, as well as DII S3–S4 KKKK and EEEE mutants, using simplified solutions containing only NaCl, to which we added variable concentrations of Ca^2+^ to the extracellular solution (Fig. 2*H*).

In the absence of extracellular Ca^2+^, WT Na_V_1.7 activated at –34 ± 3 mV, shifting to –21 ± 3 mV with 2 mM Ca^2^^+^ (Δ*V*_50_ = +13 mV), consistent with classical charge screening (Hille, 1968; Hille et al., 1975) (Fig. 2*I*). DII KKKK mutants activated at –21 ± 3 mV in 0 mM Ca^2^^+^, similar to WT in 2 mM Ca^2+^, and depolarized further to –6 ± 6 mV with 2 mM Ca^2^^+^, demonstrating additive screening effects (Fig. 2*J*, Table 6).

**Table 6:** Voltage‐dependence of activation of Na_V_1.7 KKKK and EEEE loop mutants with different extracellular calcium concentrations

Channel constructs and Ca^2+^ concentration	*V*_50_ of activation (mV)	*P*	*n* (cells)
Na_V_1.7 WT, 0 mM Ca^2+^	−34 ± 3	—	6
Na_V_1.7 WT, 2 mM Ca^2+^	−21 ± 3^*^	<0.001	5
Na_V_1.7 DII KKKK, 0 mM Ca^2+^	−21 ± 3	—	5
Na_V_1.7 DII KKKK, 2 mM Ca^2+^	−6 ± 6^*^	<0.001	6
Na_V_1.7 DII EEEE, 0 mM Ca^2+^	−33 ± 3	—	6
Na_V_1.7 DII EEEE, 2 mM Ca^2+^	−33 ± 4	0.995	10
Na_V_1.7 DII EEEE, 20 mM Ca^2+^	−28 ± 4	0.0574	9
Na_V_1.7 DII EEEE, 50 mM Ca^2+^	−3 ± 6^*^	<0.001	7

Data represented as means ± SD. Activation *V*_50_ of WT and Na_V_1.7 channel mutants compared to 0 mM Ca^2+^using unpaired *t* test or ordinary one‐way ANOVA with Dunnett's post test.

* *P*<0.05.

By contrast, DII EEEE mutants were largely insensitive to lower concentrations of extracellular Ca^2+^. In 0 mM Ca^2+^, *V*_50_ was –33 ± 3 mV, shifting minimally to –33 ± 4 mV at 2 mM and –28 ± 4 mV at 20 mM Ca^2+^. At the highest concentration, 50 mM Ca^2+^, *V*_50_ depolarized dramatically to –3 ± 6 mV, (Fig. 2*K*, Table 6). These data indicate that negatively charged loops require higher extracellular Ca^2+^ to overcome their electrostatic effects.

Together, these results support a model in which net charge in the DII S3–S4 loop shapes the electrostatic environment of the S4 voltage sensor, controlling channel activation, and where extracellular Ca^2^^+^ can modify surface charges to tune voltage sensitivity.

### Charge‐pair interactions within the channel are unlikely to cause the depolarizing shift caused by the Na_V_1.7 DII S3–S4 KKGS mutation

**Figure 3:**
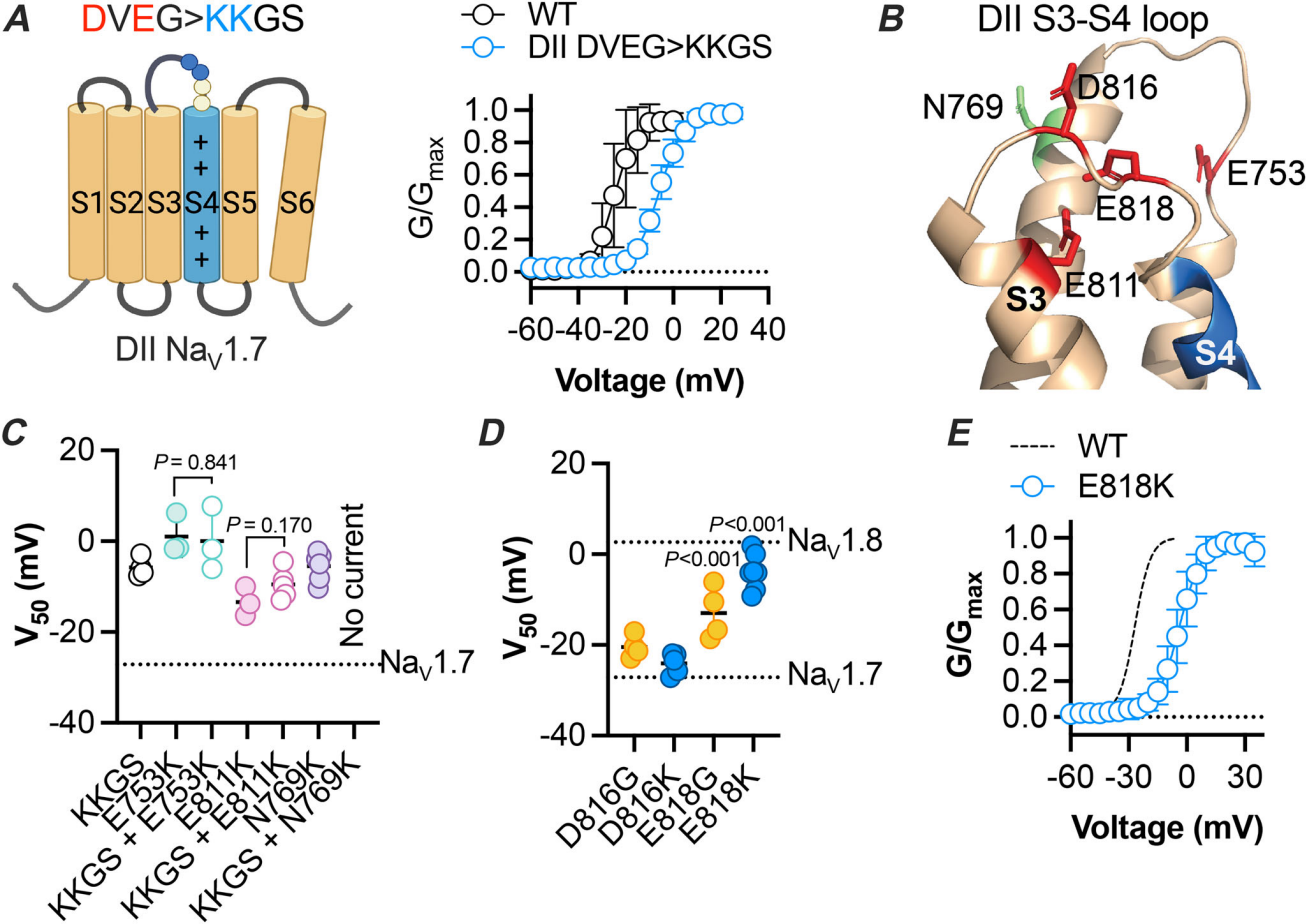
Molecular determinants of DII S3–S4 loop‐mediated regulation of Na_V_1.7 activation *A*, conductance–voltage relationship of WT Na_V_1.7 (DVEG) compared to Na_V_1.7 DII KKGS mutant (*n* = 4–6 cells). *B*, cryo‐electron microscopy structure of the human Na_V_1.7 showing Domain II (DII) (PDB ID: 6N4R), with residues potentially involved in charge‐pair interactions labelled. *C*, voltage‐dependence of activation of Na_V_1.7 with the E753K, E811K or N769K mutation, either alone or combined with the DII S3–S4 KKGS motif (*n* = 3–6 cells). Statistical significance was determined using unpaired *t* test. *D*, voltage‐dependence of activation of Na_V_1.7 mutants D816G, D816K, E818G, E818K (*n* = 4–7 cells). Statistical significance was determined using one‐way ANOVA with Dunnett's multiple comparisons test compared to wild‐type Na_V_1.7. Conductance–voltage relationship of WT Na_V_1.7 (DVEG) compared to Na_V_1.7 DII KKGS mutant (*n* = 4–7 cells). *E*, conductance–voltage (*G–V*) relationship of WT Na_V_1.7 (E818) compared to Na_V_1.7 E818K mutant (*n* = 6–7 cells).

Having established that increasing positive surface charge in the DII S3–S4 loop can depolarize Na_V_1.7 activation, we next assessed whether the naturally occurring positively charged sequence from Na_V_1.8 (KKGS) alone was enough to produce a similar effect. We introduced the KKGS substitution into Na_V_1.7, mimicking the DII S3–S4 loop of Na_V_1.8, and measured its effect on channel activation (Fig. 3*A*). This mutation caused a pronounced depolarizing shift in *V*_50_ (–6 ± 2 mV) relative to WT Na_V_1.7 (–24 ± 2 mV), consistent with the idea that the net positive charge in this loop contributes to voltage sensitivity.

To determine whether the depolarizing shift caused by the KKGS mutation could arise from new charge‐pair interactions with nearby residues, we focused on positions that normally interact with S4 gating charges during activation: E753 (S1) and E811 (S3), which form salt bridges with the first and second arginine residues (R1 and R2) in the S4 segment of DII, and N769 on S2, which hydrogen‐bonds with the third S4 arginine residue (R3) (Fig. 3*B*) (Xu et al., 2019). Each residue was individually mutated to lysine (E753K, E811K, N769K), both alone and in combination with the KKGS motif, to test whether these interactions may contribute to the observed shift in activation.

We observed no significant difference in *V*_50_ between E753K alone (1 ± 5 mV) and E753K + KKGS (0 ± 7 mV), or between E811K alone (–13 ± 3 mV) and E811K + KKGS (–10 ± 3 mV), and no current could be recorded for N769K + KKGS (Fig. 3*C*, Table 7). These results indicate that the depolarizing shift induced by the KKGS mutation are unlikely to result from new charge‐pair interactions, but instead reflects changes in the surface electrostatic environment of the DII S3–S4 loop.

**Table 7:** Voltage‐dependence of activation of Na_V_1.7 DII loop mutants

Channel constructs	*V*_50_ of activation (mV)	*P*	*n* (cells)
Na_V_1.7 DII KKGS	−6 ± 2	<0.001	4
Na_V_1.7 DII E753K	1 ± 5	—	3
Na_V_1.7 DII KKGS + E753K	0 ± 7	0.841	3
Na_V_1.7 DII E811K	−13 ± 3	—	3
Na_V_1.7 DII KKGS + E811K	−10 ± 3	0.170	5
Na_V_1.7 DII N769K	−5 ± 3	—	6
Na_V_1.7 DII KKGS + N769K	No current	—	—
Na_V_1.7 D816G	−20 ± 2	0.185	4
Na_V_1.7 D816K	−24 ± 2	0.979	5
Na_V_1.7 E818G	−13 ± 6^*^	<0.001	4
Na_V_1.7 E818K	−4 ± 4^*^	<0.001	7

Data represented as means ± SD. Activation *V*_50_ of Na_V_1.7 channel mutants compared to WT Na_V_1.7 or corresponding charge‐pair interaction using unpaired *t* test or ordinary one‐way ANOVA with Dunnett's post test.

* *P*<0.05.

### E818 in the DII S3–S4 loop is a key determinant of Na_V_1.7 activation voltage

To assess the contribution of individual charges in the DII S3–S4 loop of Na_V_1.7, we mutated two negatively charged residues closest to the S4 voltage sensors: aspartic acid D816 and glutamic acid E818. Each residue was either neutralized (D816G, E818G) or charge‐reversed to lysine (D816K, E818K).

Modification of D816 had minimal effects on activation: D816G (*V*_50_ –20 ± 2 mV) and D816K (*V*_50_ –24 ± 2 mV) were not significantly different from WT (*V*_50_ –24 ± 2 mV) (Fig. 3*D*, Table 7). By contrast, altering E818 produced a pronounced depolarizing shift in the voltage dependence of activation. Neutralization of E818 (E818G) shifted *V*_50_ to –13 ± 6 mV, whereas charge reversal (E818K) caused a further depolarizing shift to –4 ± 4 mV (Fig. 3*D* and *E*, Table 7). Together, these results identify E818 as a principal determinant of Na_V_1.7 activation voltage within the DII S3–S4 loop.

### Surface charges tune toxin‐induced modulation of Na_V_1.7 activation

In light of the observations that charged residues in the DII S3–S4 extracellular loop – the binding site of several gating‐modifier toxins – contributes to setting the activation threshold of Na_V_1.7, we next reasoned that charge screening of extracellular loop residues induced by peptide binding could contribute to the biophysical effects of toxin binding – specifically, a depolarizing shift in the voltage‐dependence of activation. Furthermore, we reasoned that the magnitude of this shift can be rationally modified by altering the overall peptide surface charge. To assess this hypothesis, we used the Na_V_1.7‐selective spider toxin Pn3a, for which extensive structure–activity data, including residues interacting with the DII S3–S4 extracellular loop, are available.

**Figure 4:**
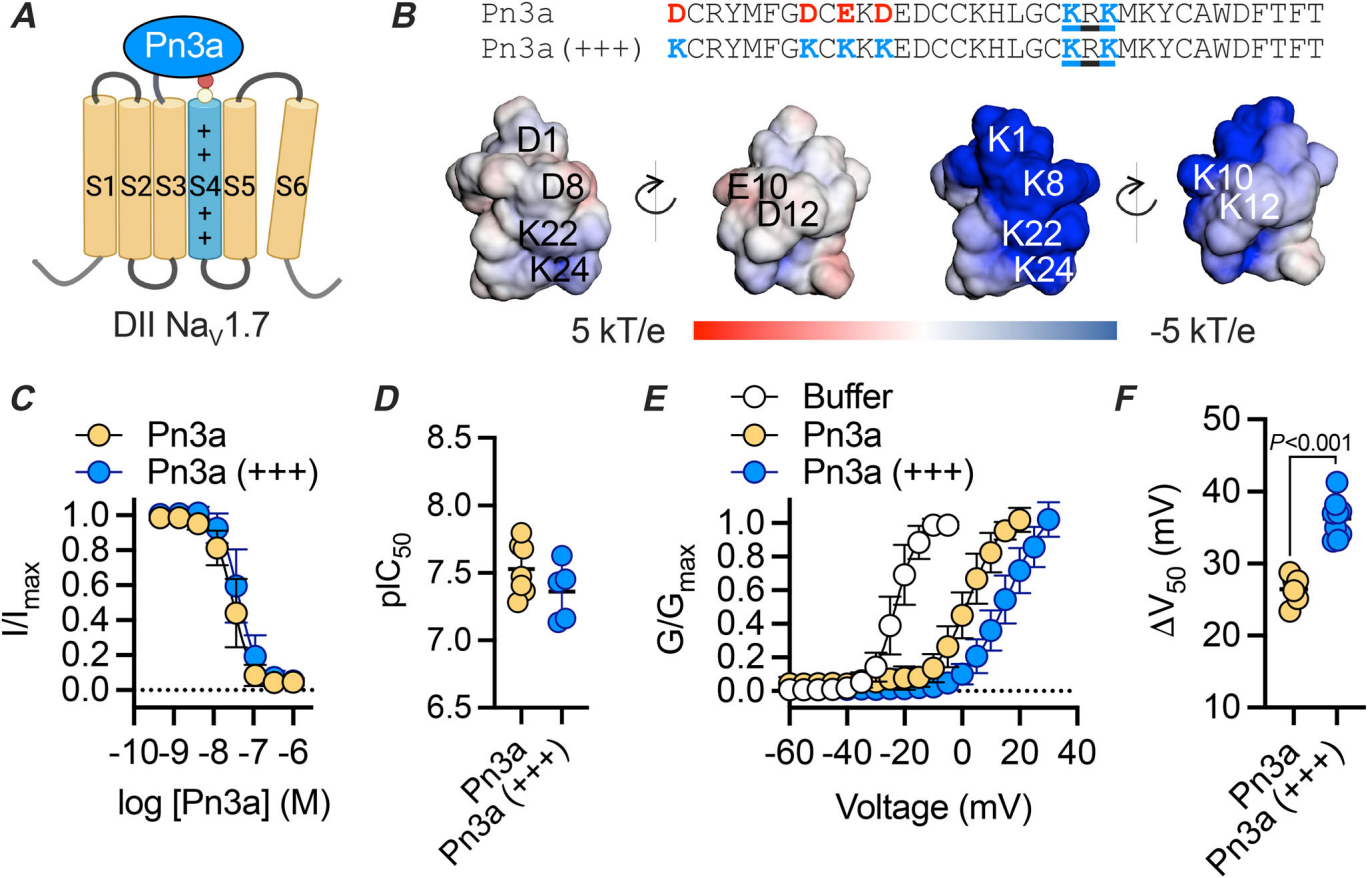
Pn3a surface charge engineering enhances its effects on Na_V_1.7 gating *A*, Pn3a binds to the DII S3–S4 extracellular loop of Na_V_1.7. *B*, sequence and surface charge distribution of wild‐type Pn3a and charge‐enhanced Pn3a. Surface charge distribution was generated by APBS software. Residues predicted to mediate channel binding are underlined. *C*, concentration–response curve of wild‐type and charge‐enhanced Pn3a at Na_V_1.7 (*n* = 5–7). *D*, peptide potency (pIC_50_) for wild‐type and charge‐enhanced Pn3a at Na_V_1.7; no significant difference was observed. *E*, conductance–voltage (*G–V*) relationship of Na_V_1.7 followed by addition of buffer, wild‐type Pn3a (1 µM) or charge‐enhanced Pn3a (1 µM); *n* = 6–7 cells. *F*, shift in activation voltage (Δ*V*_50_) for wild‐type and charge‐enhanced Pn3a relative to buffer. Statistical significance was determined using unpaired *t* test.

Like many other Na_V_ gating modifier toxins, Pn3a binds the DII S3–S4 extracellular loop and produces a depolarizing shift in channel activation (Deuis, Dekan et al., 2017) (Fig. 4*A*). Positively charged residues on Pn3a, including K22 and K24, likely form electrostatic interactions with negatively charged residues in the Na_V_1.7 DII S3–S4 loop, such as D816 and E818, suggesting that peptide surface charge may influence its gating effects (Mueller et al., 2020).

To directly test the contribution of peptide surface charge, we generated a charge‐enhanced Pn3a analogue in which the NaV‐interacting surface (including K22 and K24) was left unchanged, but in which four solvent‐exposed acidic residues (D1, D8, E10 and E13) were replaced with lysines. Whereas wild‐type Pn3a is approximately neutral (∼0 e), the charge‐enhanced analogue carries a net charge of +8 e, without altering residues predicted to mediate channel binding (Fig. 4*B*).

Indeed, potency of the charge enhanced Pn3a analogue was unchanged. Wild‐type Pn3a inhibited Na_V_1.7 with an IC_50_ of 32 nM (pIC_50_ 7.5 ± 0.2, *n* = 7), whereas the charge‐enhanced analogue had an IC_50_ of 50 nM (pIC_50_ 7.4 ± 0.2; *n* = 5; *P* = 0.185 unpaired *t* test) (Fig. 4*C* and *D*). This indicates that the charge substitutions do not alter peptide potency.

Consistent with our surface charge hypothesis, the charge‐enhanced Pn3a analogue produced a larger depolarizing shift in channel activation compared to the wild‐type peptide. Specifically, addition of wild‐type Pn3a (1 µM) caused a Δ*V*_50_ shift of 26 ± 2 mV (*n* = 6), whereas the positively charged quadruple Pn3a analogue (1 µM) produced a further depolarizing shift, with a Δ*V*_50_ shift of 36 ± 3 mV (*n* = 9; *P* < 0.001 unpaired *t* test) (Fig. 4*E*). Thus, increasing peptide surface charge enhanced the magnitude of the activation shift produced by Pn3a (Fig. 4*F*).

Together, these results show that electrostatic properties of Pn3a tune the extent of its gating modulation, such that increasing positive surface charge augments the depolarizing shift in Na_V_1.7 activation without affecting potency.

## Discussion

In this study, we identify extracellular loop charges in DII as a critical determinant of voltage‐dependent activation in Na_V_ channels. Using Na_V_1.7/Na_V_1.8 chimeras, targeted charge substitutions, extracellular charge screening, and the peptide Pn3a, we demonstrate that the DII S3–S4 extracellular loop sets the activation threshold by modulating the local electrostatic environment experienced by the S4 voltage sensor. Together, our data reveal that extracellular loop charge in DII directly shapes the electrostatic environment of the S4 voltage sensor, providing a key mechanism underlying isoform‐specific differences in Na_V_ channel activation.

Replacing the DII S3–S4 extracellular loop of Na_V_1.7 with that from Na_V_1.8 shifted activation to voltages comparable to Na_V_1.8. This indicates that a relatively small loop can exert a dominant influence on voltage sensitivity. Sequence analysis revealed a strong net positive charge in the Na_V_1.8 loop compared with the negatively charged Na_V_1.7 loop (Fig. 1*E*), suggesting an electrostatic mechanism. By contrast, substituting the native Na_V_1.7 loop into Na_V_1.8, which introduces only two negative charges, had little effect, whereas introducing a fully negative EEEE motif caused a pronounced hyperpolarizing shift toward Na_V_1.7‐like values. These results indicate that modest changes in surface charge are insufficient to overcome the intrinsically depolarized gating of Na_V_1.8, suggesting that additional structural elements contribute to its activation threshold. Consistent with this, recent studies identified that the DII S5 residues K806 and L809 formed stabilizing interactions with the DI voltage‐sensing domain, biasing Na_V_1.8 toward depolarized activation (Huang et al., 2022). Together, these observations suggest that Na_V_1.8 gating is shaped by a combination of extracellular electrostatics and interdomain coupling mechanisms.

Despite containing positively charged residues at the equivalent position within the DII S3–S4 extracellular loop (QKRS), Na_V_1.9 displays a markedly hyperpolarized activation threshold compared with Na_V_1.7 and Na_V_1.8 (Dib‐Hajj et al., 2015). However, Na_V_1.9 is also highly structurally and functionally divergent from these isoforms, with substantial sequence differences across voltage‐sensing domains and S4–S5/pore‐coupling interfaces known to influence channel activation. Its strongly hyperpolarized activation threshold is likely governed by additional structural and biophysical mechanisms that are either weak or absent in Na_V_1.7 and Na_V_1.8 channels.

Our Na_V_1.7 DII S3–S4 charge manipulations, in which insertion of four lysines (KKKK) depolarized activation and four glutamates (EEEE) hyperpolarized activation, closely mirror findings in the Kv1.7 homologue from electric fish, where analogous substitutions near the S4 segment produced similar predictable shifts (Swapna et al., 2018). These results suggest that the charge of the S3–S4 extracellular loop is a conserved determinant of voltage sensor activation across voltage‐gated ion channels. Unlike Kv channels, which are tetramers of identical subunits, Na_V_ channels contain four non‐identical voltage‐sensing domains, allowing for domain‐specific effects. Indeed, the asymmetry observed across Na_V_ domains following KKKK insertion argues against a simple, non‐specific surface charge effect and instead supports a model in which the local electrostatic environment surrounding each voltage sensor differentially tunes its energetics. In DII, positive residues in the S3–S4 loop generate a local potential that reduces the effective depolarization sensed by S4, requiring larger membrane depolarizations to trigger activation. By contrast, DI and DIII showed smaller or even opposite shifts with KKKK, reflecting domain‐specific contributions from intrinsic S4 charge distribution, loop orientation and stabilizing interactions with neighbouring helices. DIV was largely insensitive, consistent with its specialized role in coupling activation to fast inactivation rather than driving pore opening (Brake et al., 2022; Capes et al., 2013; Chanda & Bezanilla, 2002; Goldschen‐Ohm et al., 2013).

In Na_V_1.7, the more pronounced depolarizing shift observed with the KKGS substitution compared to KKKK suggests that activation is not governed solely by the number of positive charges within the DII S3–S4 loop but by the spatial presentation of charged residues relative to the S4 voltage sensor. In particular, the position, orientation and solvent exposure of individual side chains are likely critical in shaping the local electrostatic environment that stabilizes distinct voltage‐sensor conformations. Under this framework, the two lysines introduced in KKGS likely occupy positions that are particularly effective at modulating S4 movement, whereas additional lysines in KKKK may not contribute additively due to suboptimal orientation, limited exposure, or local conformational constraints within the loop. This interpretation is consistent with the distinct effects observed for individual substitutions at D816 and E818 and supports an electrostatic mechanism in which charge position and presentation, rather than number of charges alone, is a key determinant of functional outcome.

One possible structural interpretation of these findings is that positive charges introduced into the DII S3–S4 loop adjacent to the extracellular end of S4 may effectively increase the positive electrostatic environment associated with the voltage sensor. This could stabilize S4 in its resting/downward position, for example through enhanced electrostatic attraction toward the intracellular negative potential, thereby requiring stronger depolarization to drive outward S4 movement. This mechanism remains speculative in the absence of structures of the mutant channels but provides a plausible structural framework for how loop charge may tune voltage‐sensor activation.

Targeted mutations of residues within the S3–S4 loop identified E818, the negatively charged residue closest to S4, as a dominant determinant of activation voltage. Neutralization or charge reversal of E818 produced pronounced depolarizing shifts, whereas analogous modifications of D816 had minimal effect, highlighting the importance of residue position relative to the voltage sensor. While these mutations could, in principle, introduce or disrupt specific charge–charge interactions with S4 gating residues, substitution of neighbouring residues predicted to interact with S4 gating charges, including E753, E811 and N769, had little impact on activation. Collectively, these data do not support a dominant role for discrete pairwise interactions at these positions, but instead indicate that voltage sensitivity is primarily governed by the broader local electrostatic environment generated by the S3–S4 loop.

In addition to altering electrostatic properties, substitutions at E818 also changed side‐chain size and chemistry, raising the possibility that steric effects could contribute to the observed gating shifts. However, the functional effects are unlikely to be explained by altered side‐chain volume alone. Glycine is substantially smaller than glutamate, and if steric constraints were dominant, reducing side‐chain bulk would be expected to facilitate voltage‐sensor movement and favour activation. Instead, E818G produced a depolarizing shift and impaired activation. Conversely, E818K, which restores substantial side‐chain size while reversing the charge, caused an even larger depolarizing shift. Together, these findings support a model in which the effects of E818 substitutions are driven primarily by electrostatic interactions rather than steric constraints, although additional local structural contributions cannot be excluded in the absence of high‐resolution mutant channel structures.

Separately, extracellular Ca^2^^+^ experiments provided independent support for a surface charge mechanism. Increasing Ca^2^^+^ concentrations shifted Na_V_1.7 activation in a manner consistent with electrostatic screening: channels with negatively charged DII loops required higher Ca^2^^+^ to depolarize activation, whereas channels with positively charged loops were already depolarized at low Ca^2^^+^. These results indicate that Ca^2^^+^ is unlikely to act through a specific binding site in this region but instead modulates activation by screening the local electrostatic environment sensed by the S4 voltage sensor, highlighting the strong influence of clustered loop charges on voltage sensitivity. Consistent with an electrostatic screening mechanism, we have previously shown that decreasing extracellular Na^+^ concentration produces a similar effect to decreasing extracellular Ca^2^^+^ (Thapa et al., 2024).

Our Pn3a data extend these findings to gating‐modifier peptides. Although no cryo‐EM structure of the Pn3a‐Na_V_1.7 complex is currently available, previous functional and mutagenesis studies suggest that Pn3a residues K22 and K24 contribute to Na_V_1.7 binding, potentially through interactions with acidic residues in the DII S3–S4 loop, including D816 and E818 (Mueller et al., 2020), suggesting that Pn3a can depolarize activation either by neutralizing these negative charges and/or by introducing additional positive charge. The relative contributions of electrostatic interactions versus steric stabilization of the S4 voltage sensor are difficult to separate, as both mechanisms can produce similar shifts in activation (Wisedchaisri et al., 2021). Nevertheless, our results show that the magnitude of the depolarizing effect can be tuned by altering the net charge of the peptide, directly linking surface electrostatics to functional impact and highlighting modulation of peptide electrostatics as a potential strategy to enhance the activity of gating‐modifier toxins, with possible applicability to other Na_V_‐targeted peptides.

In conclusion, our study establishes that extracellular loop charge in DII, particularly the S3–S4 loop, is a key determinant of Na_V_ channel activation, acting via modulation of the local electrostatic environment around the S4 voltage sensor. This mechanism, conserved across voltage‐gated ion channels, interacts with domain‐specific structural features, and can be exploited both by natural and engineered peptides to fine‐tune channel function.

## Additional information

## Competing interests

The authors declare no competing interests.

## Author contributions

Conceptualization (A.T., L.R., H.T., A.K., J.D., I.V.); methodology (A.T., L.R., H.T., A.K., J.D., I.V.); formal analysis (A.T., A.K., J.D.); investigation (A.T., L.R., H.T., J.D.); writing – original draft (A.T., A.K., J.D.); writing – review & editing (L.R., H.T., A.K., J.D., I.V.); supervision (L.R., A.K., J.D., I.V.); funding acquisition (A.K., I.V.). All authors approved the final version of the manuscript and agree to be accountable for all aspects of the work, including ensuring that questions related to the accuracy or integrity of any part of the work are appropriately investigated and resolved. All individuals listed as authors qualify for authorship, and all those who qualify for authorship are included.

## Funding

The work was supported by the Australian Research Council Discovery Project (DP230100728) awarded to A.K. and I.V.

## Generative AI statement

ChatGPT (OpenAI, GPT‐5.3‐mini) was used in the preparation of this manuscript to assist with writing and editing only.

## Supporting information


Peer Review History

## Data Availability

All data supporting the findings of this study are provided in the manuscript.

## References

[tjp70727-bib-0001] Ahern, C. A. , Payandeh, J. , Bosmans, F. , & Chanda, B. (2016). The hitchhiker's guide to the voltage‐gated sodium channel galaxy. Journal of General Physiology, 147(1), 1–24. 26712848 10.1085/jgp.201511492 PMC4692491

[tjp70727-bib-0002] Bosmans, F. , Martin‐Eauclaire, M. F. , & Swartz, K. J. (2008). Deconstructing voltage sensor function and pharmacology in sodium channels. Nature, 456(7219), 202–208. 19005548 10.1038/nature07473 PMC2587061

[tjp70727-bib-0003] Brake, N. , Mancino, A. S. , Yan, Y. , Shimomura, T. , Kubo, Y. , Khadra, A. , & Bowie, D. (2022). Closed‐state inactivation of cardiac, skeletal, and neuronal sodium channels is isoform specific. Journal of General Physiology 154(7), e202112921. 35612552 10.1085/jgp.202112921 PMC9136305

[tjp70727-bib-0004] Capes, D. L. , Goldschen‐Ohm, M. P. , Arcisio‐Miranda, M. , Bezanilla, F. , & Chanda, B. (2013). Domain IV voltage‐sensor movement is both sufficient and rate limiting for fast inactivation in sodium channels. Journal of General Physiology, 142(2), 101–112. 23858005 10.1085/jgp.201310998 PMC3727307

[tjp70727-bib-0005] Chanda, B. , & Bezanilla, F. (2002). Tracking voltage‐dependent conformational changes in skeletal muscle sodium channel during activation. Journal of General Physiology, 120(5), 629–645. 12407076 10.1085/jgp.20028679 PMC2229551

[tjp70727-bib-0006] Cukierman, S. , Zinkand, W. C. , French, R. J. , & Krueger, B. K. (1988). Effects of membrane surface charge and calcium on the gating of rat brain sodium channels in planar bilayers. Journal of General Physiology, 92(4), 431–447. 2849628 10.1085/jgp.92.4.431 PMC2228909

[tjp70727-bib-0007] Deuis, J. R. , Dekan, Z. , Wingerd, J. S. , Smith, J. J. , Munasinghe, N. R. , Bhola, R. F. , Imlach, W. L. , Herzig, V. , Armstrong, D. A. , Rosengren, K. J. , Bosmans, F. , Waxman, S. G. , Dib‐Hajj, S. D. , Escoubas, P. , Minett, M. S. , Christie, M. J. , King, G. F. , Alewood, P. F. , Lewis, R. J. , … Vetter, I. (2017). Pharmacological characterisation of the highly NaV1.7 selective spider venom peptide Pn3a. Scientific Reports, 7, 40883. 28106092 10.1038/srep40883 PMC5247677

[tjp70727-bib-0008] Deuis, J. R. , Mueller, A. , Israel, M. R. , & Vetter, I. (2017). The pharmacology of voltage‐gated sodium channel activators. Neuropharmacology, 127, 87–108. 28416444 10.1016/j.neuropharm.2017.04.014

[tjp70727-bib-0009] Deuis, J. R. , Ragnarsson, L. , Robinson, S. D. , Dekan, Z. , Chan, L. , Jin, A. H. , Tran, P. , McMahon, K. L. , Li, S. , Wood, J. N. , Cox, J. J. , King, G. F. , Herzig, V. , & Vetter, I. (2021). The tarantula venom peptide Eo1a binds to the domain II S3‐S4 extracellular loop of voltage‐gated sodium channel Na(V)1.8 to enhance activation. Frontiers in Pharmacology, 12, 789570. 35095499 10.3389/fphar.2021.789570 PMC8795738

[tjp70727-bib-0010] Dib‐Hajj, S. D. , Black, J. A. , & Waxman, S. G. (2015). NaV1.9: A sodium channel linked to human pain. Nature Reviews Neuroscience, 16(9), 511–519. 26243570 10.1038/nrn3977

[tjp70727-bib-0011] Goldschen‐Ohm, M. P. , Capes, D. L. , Oelstrom, K. M. , & Chanda, B. (2013). Multiple pore conformations driven by asynchronous movements of voltage sensors in a eukaryotic sodium channel. Nature Communications, 4, 1350. 10.1038/ncomms2356 PMC3562458 23322038

[tjp70727-bib-0012] Hille, B. (1968). Charges and potentials at the nerve surface. Divalent ions and pH. Journal of General Physiology, 51(2), 221–236. 10.1085/jgp.51.2.221 PMC2201126 5641636

[tjp70727-bib-0013] Hille, B. , Woodhull, A. M. , & Shapiro, B. I. (1975). Negative surface charge near sodium channels of nerve: Divalent ions, monovalent ions, and pH. Philosophical Transactions of the Royal Society of London‐Series B: Biological Sciences, 270(908), 301–318. 238230 10.1098/rstb.1975.0011

[tjp70727-bib-0014] Huang, X. , Jin, X. , Huang, G. , Huang, J. , Wu, T. , Li, Z. , Chen, J. , Kong, F. , Pan, X. , & Yan, N. (2022). Structural basis for high‐voltage activation and subtype‐specific inhibition of human Na(v)1.8. Proceedings of the National Academy of Sciences of the United States of America, 119(30), e2208211119. 35858452 10.1073/pnas.2208211119 PMC9335304

[tjp70727-bib-0015] Jurrus, E. , Engel, D. , Star, K. , Monson, K. , Brandi, J. , Felberg, L. E. , Brookes, D. H. , Wilson, L. , Chen, J. , Liles, K. , Chun, M. , Li, P. , Gohara, D. W. , Dolinsky, T. , Konecny, R. , Koes, D. R. , Nielsen, J. E. , Head‐Gordon, T. , Geng, W. , … Baker, N. A. (2018). Improvements to the APBS biomolecular solvation software suite. Protein Science, 27(1), 112–128. 28836357 10.1002/pro.3280 PMC5734301

[tjp70727-bib-0016] Köster, P. A. , Leipold, E. , Tigerholm, J. , Maxion, A. , Namer, B. , Stiehl, T. , & Lampert, A. (2025). Nociceptor sodium channels shape subthreshold phase, upstroke, and shoulder of action potentials. Journal of General Physiology 157(2), e202313526. 39836077 10.1085/jgp.202313526 PMC11748974

[tjp70727-bib-0017] Mueller, A. , Dekan, Z. , Kaas, Q. , Agwa, A. J. , Starobova, H. , Alewood, P. F. , Schroeder, C. I. , Mobli, M. , Deuis, J. R. , & Vetter, I. (2020). Mapping the molecular surface of the analgesic Na(V)1.7‐selective peptide Pn3a reveals residues essential for membrane and channel interactions. ACS Pharmacology & Translational Science, 3(3), 535–546. 32566918 10.1021/acsptsci.0c00002 PMC7296542

[tjp70727-bib-0018] Payandeh, J. , Scheuer, T. , Zheng, N. , & Catterall, W. A. (2011). The crystal structure of a voltage‐gated sodium channel. Nature, 475(7356), 353–358. 21743477 10.1038/nature10238 PMC3266868

[tjp70727-bib-0019] Rush, A. M. , Cummins, T. R. , & Waxman, S. G. (2007). Multiple sodium channels and their roles in electrogenesis within dorsal root ganglion neurons. The Journal of Physiology, 579(Pt 1), 1–14. 17158175 10.1113/jphysiol.2006.121483 PMC2075388

[tjp70727-bib-0020] Swapna, I. , Ghezzi, A. , York, J. M. , Markham, M. R. , Halling, D. B. , Lu, Y. , Gallant, J. R. , & Zakon, H. H. (2018). Electrostatic tuning of a potassium channel in electric fish. Current Biology, 28(13), 2094–2102.e5. 29937349 10.1016/j.cub.2018.05.012 PMC6067922

[tjp70727-bib-0021] Thapa, A. , Beh, J. H. , Robinson, S. D. , Deuis, J. R. , Tran, H. , Vetter, I. , & Keramidas, A. (2024). A venom peptide‐induced Na(V) channel modulation mechanism involving the interplay between fixed channel charges and ionic gradients. Journal of Biological Chemistry, 300(10), 107757. 39260690 10.1016/j.jbc.2024.107757 PMC11470524

[tjp70727-bib-0022] Vijayaragavan, K. , O'Leary, M. E. , & Chahine, M. (2001). Gating properties of Na(v)1.7 and Na(v)1.8 peripheral nerve sodium channels. Journal of Neuroscience, 21(20), 7909–7918. 11588164 10.1523/JNEUROSCI.21-20-07909.2001 PMC6763856

[tjp70727-bib-0023] Wisedchaisri, G. , Tonggu, L. , Gamal El‐Din, T. M. , McCord, E. , Zheng, N. , & Catterall, W. A. (2021). Structural basis for high‐affinity trapping of the Na(V)1.7 channel in its resting state by tarantula toxin. Molecular Cell, 81(1), 38–48.e4. 33232657 10.1016/j.molcel.2020.10.039 PMC8043720

[tjp70727-bib-0024] Xu, H. , Li, T. , Rohou, A. , Arthur, C. P. , Tzakoniati, F. , Wong, E. , Estevez, A. , Kugel, C. , Franke, Y. , Chen, J. , Ciferri, C. , Hackos, D. H. , Koth, C. M. , & Payandeh, J. (2019). Structural basis of Nav1.7 inhibition by a gating‐modifier spider toxin. Cell, 176(5), 702–715.e714. 30661758 10.1016/j.cell.2018.12.018

