# Supplementary material for: Electrostatic tuning of NaV channel activation by the domain II S3–S4 extracellular loop
Source: J Physiol. 2026 Jul 25;604(17):7210–24. doi: 10.1113/JP291286 (PMC13532856; doi:10.1113/JP291286)
Supplement: Supplementary file 1 [file TJP-604-7210-s001.pdf]

The Journal of Physiology

Electrostatic Tuning of Na_V Channel Activation by the Domain II S3–S4 Extracellular Loop

Ashvriya Thapa, Lotten Ragnarsson, Hue Tran, Angelo Keramidas, Jennifer R Deuis, and Irina Vetter
DOI: 10.1113/JP291286

Corresponding author(s): Jennifer Deuis (j.deuis@uq.edu.au)

The following individual(s) involved in review of this submission have agreed to reveal their identity: Ben Corry (Referee #2)

Review Timeline:

Submission Date:	27-Feb-2026
Editorial Decision:	28-Apr-2026
Revision Received:	11-Jun-2026
Accepted:	22-Jun-2026

Senior Editor: Peying Fong

Reviewing Editor: Theodore Cummins

Transaction Report:

(Note: With the exception of the correction of typographical or spelling errors that could be a source of ambiguity, letters and reports are not edited. Depending on transfer agreements, referee reports obtained elsewhere may or may not be included in this compilation. Referee reports are anonymous unless the Referee chooses to sign their reports.)

Dear Dr Deuis,

Re: JP-RP-2026-291286 "**Electrostatic Tuning of Na_V Channel Activation by the Domain II S3-S4 Extracellular Loop**" by Ashvriya Thapa, Lotten Ragnarsson, Hue Tran, Angelo Keramidas, Jennifer R Deuis, and Irina Vetter

Thank you for submitting your manuscript to The Journal of Physiology. It has been assessed by a Reviewing Editor and by 2 expert referees and we are pleased to tell you that it is acceptable for publication following satisfactory revision.

The referee reports are copied at the end of this email.

Please address all the points raised and incorporate all requested revisions or explain in your Response to Referees why a change has not been made. We hope you will find the comments helpful and that you will be able to return your revised manuscript within 4 weeks. If you require longer than this, please contact journal staff: jp@physoc.org.

Your revised manuscript should be submitted online using the link in your Author Tasks Link Not Available. This link is accessible via your account as Corresponding Author; it is not available to your co-authors. If this presents a problem, please contact journal staff (jp@physoc.org). Image files from the previous version are retained on the system. Please ensure you replace or remove any files that are being revised.

If you do not wish to submit a revised version of your manuscript, you must inform our journal staff (jp@physoc.org) or reply to this email to request withdrawal. Please note that a manuscript must be formally withdrawn from the peer review process at one journal before it may be submitted to another journal.

TRANSPARENT PEER REVIEW POLICY: To improve the transparency of its peer review process The Journal of Physiology publishes online, as supporting information, the peer review history of all articles accepted for publication. Readers will have access to decision letters, including Editors' comments and referee reports, for each version of the manuscript, as well as any author responses to peer review comments. Referees can decide whether or not they wish to be named on the peer review history document.

ABSTRACT FIGURES: Authors are expected to use The Journal's premium BioRender account to create/redraw their Abstract Figures. Information on how to access this account is here: https://physoc.onlinelibrary.wiley.com/journal/14697793/biorender-access.

This will enable Authors to create and download high-resolution figures. If authors have used the free BioRender service, they can use the instructions provided in the link above to download a high-resolution version suitable for publication.

The link provided should only be used for the purposes of this submission. Authors will be charged for figures created on this account if they are not related to this manuscript submission.

LANGUAGE EDITING AND SUPPORT FOR PUBLICATION: If you would like help with English language editing, or other article preparation support, Wiley Editing Services offers expert help, including English Language Editing, as well as translation, manuscript formatting, and figure formatting at www.wileyauthors.com/eeo/preparation. You can also find resources for Preparing Your Article for general guidance about writing and preparing your manuscript at www.wileyauthors.com/eeo/prepresources.

RIGOUR AND REPRODUCIBILITY: We recommend authors consult The Journal's Rigour and Reproducibility requirements to ensure that their manuscript is suitable for publication: https://physoc.onlinelibrary.wiley.com/pb-assets/hub-assets/physoc/documents/TJP-Rigour-and-Reproducibility-Requirements.pdf

REVISION CHECKLIST:

Check that your Methods section conforms to journal policy: https://jp.msubmit.net/cgi-bin/main.plex?form_type=display_requirements#methods

Check that data presented conforms to the statistics policy: https://jp.msubmit.net/cgi-bin/main.plex?form_type=display_requirements#statistics

Upload a full Response to Referees file. To create your 'Response to Referees' copy all the reports, including any comments from the Senior and Reviewing Editors, into a Microsoft Word, or similar, file and respond to each point, using font or background colour to distinguish comments and responses and upload as the required file type.

Please upload two versions of your manuscript text: one with all relevant changes highlighted and one clean version with no changes tracked. The manuscript file should include all tables and figure legends, but each figure/graph should be uploaded as separate, high-resolution files. The journal is now integrated with Wiley's Image Checking service. For further details, see: https://www.wiley.com/en-us/network/publishing/research-publishing/trending-stories/upholding-image-integrity-wileys-image-screening-service

You may also upload:

- 'Potential Cover Art' for consideration as the issue's cover image
- Appropriate Supporting Information (Video, audio or data set: see https://jp.msubmit.net/cgi-bin/main.plex?form_type=display_requirements#supp).

We look forward to receiving your revised submission.

If you have any queries, please reply to this email and we will be pleased to advise.

Yours sincerely,

Peying Fong
Senior Editor
The Journal of Physiology

REQUIRED ITEMS

- Author photo and profile. First or joint first authors are asked to provide a short biography (no more than 100 words for one author or 150 words in total for joint first authors) and a portrait photograph. These should be uploaded and clearly labelled together in a Word document with the revised version of the manuscript. See Information for Authors for further details.

- The reference list must be in alphabetical order, rather than numbered, to comply with our Journal format.

- Your manuscript must include a complete Additional Information section, including:
(1) author contributions,
(2) acknowledgements.

- Papers must comply with the Statistics Policy: https://jp.msubmit.net/cgi-bin/main.plex?form_type=display_requirements#statistics.

In summary:

- 'n' clearly defined (e.g. x cells from y slices in z animals) in the Methods. Authors should be mindful of pseudoreplication.

- All relevant 'n' values must be clearly stated in the main text, figures and tables.

- The most appropriate summary statistic (e.g. mean or median and standard deviation) must be used. Standard Error of the Mean (SEM) alone is not permitted.

- Exact p values must be stated. Authors must not use 'greater than' or 'less than'. Exact p values must be stated to three significant figures even when 'no statistical significance' is claimed.

- Please include an Abstract Figure file and an Abstract Figure legend. An appropriate figure legend, which should not exceed 150 words in length, should be included in the main manuscript file. The Abstract Figure is a piece of artwork designed to give readers an immediate understanding of the research and should summarise the main conclusions. If possible, the image should be easily 'readable' from left to right or top to bottom. It should show the physiological relevance of the manuscript so readers can assess the importance and content of its findings. Abstract Figures should not merely recapitulate other figures in the manuscript. Please try to keep the diagram as simple as possible and without superfluous information that may distract from the main conclusion(s). Abstract Figures must be provided by authors no later than the revised manuscript stage and should be uploaded as a separate file during online submission labelled as File Type 'Abstract Figure'. Please also ensure that you include the figure legend in the main article file. All Abstract Figures should be created using BioRender. Authors should use The Journal's premium BioRender account to export high-resolution images. Details on how to use and access the premium account are included as part of this email.

EDITOR COMMENTS

Reviewing Editor:

Comments for Authors to ensure the paper complies with the Statistics Policy:
The tables should include the number of observations and statistics should be presented as mean +/- SD, not SEM. Precise p values should be provided to three significant figures per journal policy. The only exception to this is if p is less than 0.001, in which case '<' is permitted.

Comments to the Author:
In this study the authors investigate the roles of extracellular surface charges in the extracellular loops of sodium channel voltage-sensing modules on the voltage-dependent channel gating. They show that the surface charge near domain II shapes the local electrostatic environment around the voltage sensing elements and dictates differences between Nav1.7 and Nav1.8 sodium channel activation.

Both reviewers found that the study had merit and was of interest to the readers of the Journal of Physiology. Reviewer 2 had specific suggestions for clarifying the discussion of the possible mechanistic implications.

In addition to these suggestions, the authors should consider also the following issues:

1) Nav1.7 is known to have a splice variant that impacts the number of negative charges in the domain II S3-S4 linker. This should be briefly discussed.

2) Nav1.9 channels, like Nav1.8 channels, have two positively charged residues in the domain II S3-S4 linker, yet Nav1.9 has a more negative voltage-dependence of activation than either Nav1.8 or Nav1.7. Does the current data provide any insight into this difference?

Senior Editor:

Comments for Authors to ensure the paper complies with the Statistics Policy:
In preparing your revised manuscript, I commend you to The Journal of Physiology's published Statistics Policy. In their comments, both Referee 1 and the Reviewing Editor summarize specific aspects that require your attention.

Comments to the Author:
Review of your manuscript, "Electrostatic Tuning of NaV Channel Activation by the Domain II S3-S4 Extracellular Loop" is now complete. Two Expert Referees and a Reviewing Editor concur on the potential impact of your study and remark on the high quality of this initial submission. In reviewing these reports, I feel you will be able to address most points raised readily. A few aspects will require deeper thought experiments; for example, both Referees identify potential for structural ramifications of altering loop residue charges that perhaps ultimately will impact mechanism (Ref. 1, point 4; Ref. 2, paragraph 4). In addition, Referee 2 offers an approach for further strengthening the argument for electrostatic calcium screening. The Reviewing Editor also identifies additional points that you might find useful in deepening the interpretation and discussion of your study.

Thank you for submitting your work to The Journal of Physiology. I look forward to receiving your revised manuscript.

REFEREE COMMENTS

Referee #1:

The manuscript by Thapa et al. investigates how the local electrostatic environment of the extracellular loops, specifically within Domain II (DII), influences the activation of Nav1.7 and Nav1.8. Using a combination of chimeras and site-directed mutagenesis, the authors demonstrate that positive charges in the DII S3-S4 loop depolarize channel activation, while negative charges (specifically E818) hyperpolarize it. They further show that these effects are modulated by extracellular Ca^{2+} screening and an engineered toxin Pn3a (+++). The charge-enhanced toxin analogue does not alter potency but significantly increases gating-modifier efficacy. Overall, this is a technically sound and conceptually impactful study. The data provides a clear mechanistic link between extracellular loop charge and the voltage-dependence of sodium channel activation, offering significant implications for both basic biophysics and the rational design of therapeutic ligands. I have no

major concerns, but suggest the following minor points for clarification:

1, On page 6, line 113, there is a typo in the word "whereas". Please correct it.

2, In the sections describing automated and conventional patch-clamp electrophysiology, series resistance is reported between 5-12 MΩ. Despite 60-70% compensation, large sodium currents may still induce significant voltage errors. Please provide a family of representative Nav1.7 and Nav1.8 current traces to clarify the quality of voltage control.

3, Four acidic residues in the Pn3a toxin were replaced with positive residues. This charge-enhanced data is excellent as it separates binding affinity (pIC50) from functional efficacy (V50). Did this quadruple mutation significantly affect the correct folding of the peptide? Is there experiment data to support this conclusion?

4, On page 15, line 408, the E818G mutation neutralizes the charge but also substantially reduces the side chain volume. Please discuss whether the observed shift could be partially attributed to this structural change rather than purely electrostatic effects.

5, Please specify the voltage at which the concentration-response curves of Pn3a and its analogue were measured. Given that they differentially shift the voltage dependence of Nav1.7 activation (Fig. 4E), the potency may vary at voltages more positive than 0 mV.

6, Per journal guidelines, all data should be reported as mean {plus minus} SD rather than mean {plus minus} SEM. Additionally, please provide exact p-values for all comparisons.

7, In Tables 3 and 4, please include the number of experimental cells (n) for each construct, including WT and mutants.

8, In Figure 4B (below), the residues are labeled with the same names for both Pn3a and Pn3a (+++). Please update the labels to correctly distinguish between the two peptides.

9, Please use the abbreviation "Na_V" consistently instead of "Nav" across all four figures.

Referee #2:

This manuscript examines the structural characteristics that tune the voltage sensitivity of sodium channels. By comparing the sequence and activation thresholds of Nav1.7 and Nav1.8 they are able to pinpoint the importance of charges in the domain II voltage sensor extracellular loops, which is tested through careful mutagenesis studies. They propose an electrostatic mechanism of this effect tested through the introduction of calcium and by tuning the charge on peptides that bind to this region of the protein. Overall, this is an excellent paper. It is very clearly written and provides valuable information that improves our understanding of voltage gated channels. The experiments are appropriate and well conducted. I have a few comments and suggestions that mostly relate to the mechanistic interpretation.

It is proposed that the impact of the loop charges on the threshold for activation is via an electrostatic effect. The impact of Ca2+ and the Pn3a toxin support this. However, there is some data that is harder to rationalise. Most notably, the introduction of 2 positive residues to Nav1.7 to reproduce the Nav1.8 sequence DVEG -> KKGS has the same or greater effect as changing DVEG -> KKKK. The electrostatic interpretation should suggest that the threshold would be more depolarised with KKKK than KKGS. How is this rationalised? I feel this point needs to be more carefully considered.

The effect of Ca2+ is said to be through electrostatic screening rather than specific binding. This is very likely given the data, but I was not entirely convinced that binding could be ruled out. One way to test this would be to show that increasing Na+ concentration can have the same effect as adding Ca2+ - noting that four times as much sodium would be required to have the same screening effect.

An alternative mechanistic explanation is that these mutations effectively increase the charge on the S4 helix as they are on the loop region adjacent to the top of the helix. Increased charge would yield a strong attraction to the negative interior of the cell, meaning that the cytoplasm has to depolarise further to induce S4 movement. I am not sure this is contrary to the electrostatic proposal given in the paper as both relate to electrostatic interactions, this, however, gives it a more structural emphasis. The authors have been careful to be factual and not provide unwarranted speculation, but I am at least intrigued to consider how the electrostatic tuning works at a structural level.

Minor comments:

Line 111 : Given that Nav can contain beta subunits, I recommend changing the wording of

"NaV channels are composed of a single alpha subunit organized" to "Nav Contain a single alpha subunit ...

Figure 3B, I recommend adding a label to be clear this is DII

Line 395: I recommend softening the wording about charged pair interactions. It is possible that such interactions exist between residues not considered in this analysis.

Line 426: It would nice to be clear about what is known structurally for the Pn3a - Nav1.7 interaction. Even a statement such as "while there is not cryo-EM structure of the Pn3a - Nav1.7 complex previous mutation studies suggest that K22 and K24 directly interact with Nav1.7 possible by binding to D816 and E818" and include the citation.

END OF COMMENTS

REQUIRED ITEMS

- Author photo and profile. First or joint first authors are asked to provide a short biography (no more than 100 words for one author or 150 words in total for joint first authors) and a portrait photograph. These should be uploaded and clearly labelled together in a Word document with the revised version of the manuscript.
See Information for Authors for further details.

This has been added.

- The reference list must be in alphabetical order, rather than numbered, to comply with our Journal format.

Referencing formatting updated to comply with journal format.

- Your manuscript must include a complete Additional Information section, including:
(1) author contributions,
(2) acknowledgements.

Author contributions have been added. No further acknowledgements to add.

Added

- Papers must comply with the Statistics Policy: https://jp.msubmit.net/cgi-bin/main.plex?form_type=display_requirements#statistics.

In summary:

- 'n' clearly defined (e.g. x cells from y slices in z animals) in the Methods. Authors should be mindful of pseudoreplication.

- All relevant 'n' values must be clearly stated in the main text, figures and tables.

- The most appropriate summary statistic (e.g. mean or median and standard deviation) must be used. Standard Error of the Mean (SEM) alone is not permitted.

- Exact p values must be stated. Authors must not use 'greater than' or 'less than'. Exact p values must be stated to three significant figures even when 'no statistical significance' is claimed.

Paper has been updated to comply with the statistics policy. 'n' has been defined in the

methods as number of cells. All ‘n’ values are provided in figure legends/tables/text where applicable. Mean and standard deviation (SD) are now used for the reporting of all numerical results. Exact p values are now provided in tables.

- Please include an Abstract Figure file and an Abstract Figure legend. An appropriate figure legend, which should not exceed 150 words in length, should be included in the main manuscript file. The Abstract Figure is a piece of artwork designed to give readers an immediate understanding of the research and should summarise the main conclusions. If possible, the image should be easily 'readable' from left to right or top to bottom. It should show the physiological relevance of the manuscript so readers can assess the importance and content of its findings. Abstract Figures should not merely recapitulate other figures in the manuscript. Please try to keep the diagram as simple as possible and without superfluous information that may distract from the main conclusion(s). Abstract Figures must be provided by authors no later than the revised manuscript stage and should be uploaded as a separate file during online submission labelled as File Type 'Abstract Figure'. Please also ensure that you include the figure legend in the main article file. All Abstract Figures should be created using BioRender. Authors should use The Journal's premium BioRender account to export high-resolution images. Details on how to use and access the premium account are included as part of this email.

This has now been included.

EDITOR COMMENTS

Reviewing Editor:

Comments for Authors to ensure the paper complies with the Statistics Policy:
The tables should include the number of observations and statistics should be presented as mean +/- SD, not SEM. Precise p values should be provided to three significant figures per journal policy. The only exception to this is if p is less than 0.001, in which case '<' is permitted.

This has all been updated to comply with journal policy.

Comments to the Author:
In this study the authors investigate the roles of extracellular surface charges in the extracellular loops of sodium channel voltage-sensing modules on the voltage-

dependent channel gating. They show that the surface charge near domain II shapes the local electrostatic environment around the voltage sensing elements and dictates differences between Nav1.7 and Nav1.8 sodium channel activation.

Both reviewers found that the study had merit and was of interest to the readers of the Journal of Physiology. Reviewer 2 had specific suggestions for clarifying the discussion of the possible mechanistic implications.

In addition to these suggestions, the authors should consider also the following issues:

1) Nav1.7 is known to have a splice variant that impacts the number of negative charges in the domain II S3-S4 linker. This should be briefly discussed.

In Na_V1.7, the most well-characterised splice variation affecting extracellular loop composition is the mutually exclusive inclusion of exons 5A and 5N within the domain I S3–S4 linker. To date, no splice variant has been identified in the domain II S3–S4 linker that demonstrably alters its negative charge content in the manner suggested. Given that our data indicate domain II is the primary determinant of NaV1.7 activation, we therefore consider discussion of such a variant to be outside the scope of the present study.

2) Nav1.9 channels, like Nav1.8 channels, have two positively charged residues in the domain II S3-S4 linker, yet Nav1.9 has a more negative voltage-dependence of activation than either Nav1.8 or Nav1.7. Does the current data provide any insight into this difference?

We thank the reviewer for this insightful question. Our current data indicate that the presence of positively charged residues within the DII S3–S4 loop alone is not sufficient to predict activation voltage, and therefore cannot account for the marked hyperpolarised activation of Na_V1.9. This suggests that additional structural determinants, beyond extracellular loop charge, make a dominant contribution to setting the activation threshold in this isoform. Given the extensive sequence and functional divergence of Na_V1.9 from Na_V1.7 and Na_V1.8, including differences across voltage-sensing domains and S4–S5/pore-coupling interfaces, it is likely that multiple distributed structural features collectively shift its voltage dependence. Robust heterologous expression of Na_V1.9 remains challenging, limiting direct experimental dissection of individual contributions from the S3–S4 loop. We have therefore expanded the Discussion to reflect that Na_V1.9 gating is unlikely to be governed by the same extracellular loop charge effects defined in this study, but instead reflects isoform-specific structural adaptations across the channel.

Added discussion:

"Despite containing positively charged residues at the equivalent position within the DII S3–S4 extracellular loop (QKRS), $Na_V1.9$ displays a markedly hyperpolarised activation threshold compared with $Na_V1.7$ and $Na_V1.8$ (Dib-Hajj *et al.*, 2015). However, $Na_V1.9$ is also highly structurally and functionally divergent from these isoforms, with substantial sequence differences across voltage-sensing domains and S4–S5/pore-coupling interfaces known to influence channel activation. Its strongly hyperpolarised activation threshold is likely governed by additional structural and biophysical mechanisms that are either weak or absent in $Na_V1.7$ and $Na_V1.8$ channels."

Senior Editor:

Comments for Authors to ensure the paper complies with the Statistics Policy:
In preparing your revised manuscript, I commend you to The Journal of Physiology's published Statistics Policy. In their comments, both Referee 1 and the Reviewing Editor summarize specific aspects that require your attention.

Comments to the Author:
Review of your manuscript, "Electrostatic Tuning of NaV Channel Activation by the Domain II S3-S4 Extracellular Loop" is now complete. Two Expert Referees and a Reviewing Editor concur on the potential impact of your study and remark on the high quality of this initial submission. In reviewing these reports, I feel you will be able to address most points raised readily. A few aspects will require deeper thought experiments; for example, both Referees identify potential for structural ramifications of altering loop residue charges that perhaps ultimately will impact mechanism (Ref. 1, point 4; Ref. 2, paragraph 4). In addition, Referee 2 offers an approach for further strengthening the argument for electrostatic calcium screening. The Reviewing Editor also identifies additional points that you might find useful in deepening the interpretation and discussion of your study.

Thank you for submitting your work to The Journal of Physiology. I look forward to receiving your revised manuscript.

REFEREE COMMENTS

Referee #1:

The manuscript by Thapa et al. investigates how the local electrostatic environment of the extracellular loops, specifically within Domain II (DII), influences the activation of Nav1.7 and Nav1.8. Using a combination of chimeras and site-directed mutagenesis, the authors demonstrate that positive charges in the DII S3-S4 loop depolarize channel activation, while negative charges (specifically E818) hyperpolarize it. They further show that these effects are modulated by extracellular Ca2+ screening and an engineered toxin Pn3a (+++). The charge-enhanced toxin analogue does not alter potency but significantly increases gating-modifier efficacy. Overall, this is a technically sound and conceptually impactful study. The data provides a clear mechanistic link between extracellular loop charge and the voltage-dependence of sodium channel activation, offering significant implications for both basic biophysics and the rational design of therapeutic ligands. I have no major concerns, but suggest the following minor points for clarification:

1, On page 6, line 113, there is a typo in the word "whereas". Please correct it.

We have corrected this typo.

2, In the sections describing automated and conventional patch-clamp electrophysiology, series resistance is reported between 5-12 MΩ. Despite 60-70% compensation, large sodium currents may still induce significant voltage errors. Please provide a family of representative Nav1.7 and Nav1.8 current traces to clarify the quality of voltage control.

Please see below sample representative IV traces obtained for WT $Na_V1.7$ and WT $Na_V1.8$. Only traces from -80 to 50 mV at 10 mV intervals are shown for clarity. The red traces indicate a test pulse of -20 mV for $Na_V1.7$ and 10 mV for $Na_V1.8$. To minimise series resistance errors, only cells with currents that ranged between 1-5 nA were selected. This statement has now been added to the methods.

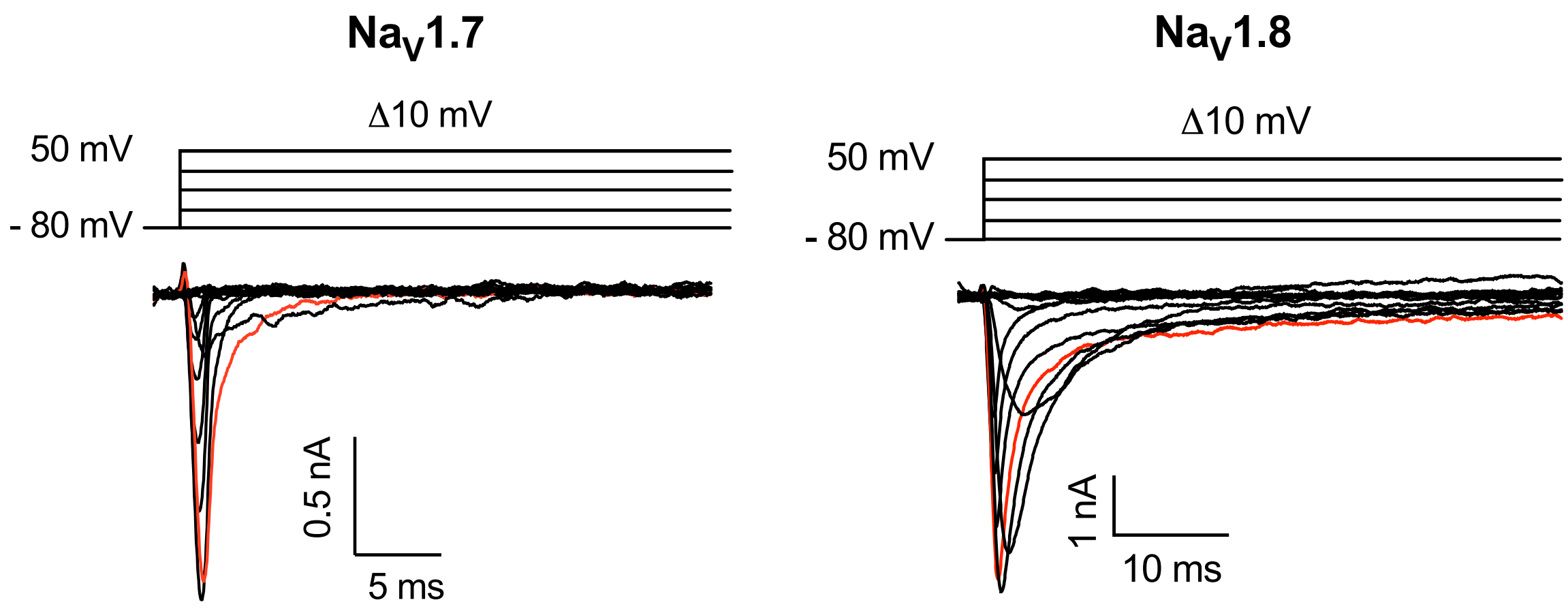

3, Four acidic residues in the Pn3a toxin were replaced with positive residues. This charge-enhanced data is excellent as it separates binding affinity (pIC50) from functional efficacy (V50). Did this quadruple mutation significantly affect the correct folding of the peptide? Is there experiment data to support this conclusion?

The quadruple mutant peptide folded correctly, based on the following experimental evidence: i) potency of the peptide was unaffected. Pn3a loses activity unless the disulfides are formed correctly, hence any peptide that retains activity is correctly folded. ii) elution peaks. The mutant peptide eluted as two peaks during RP-HPLC purification (see chromatograms below), consistent with the behaviour of correctly folded Pn3a, which generates two rapidly interconverting conformers (PMID: ***37521635)***.

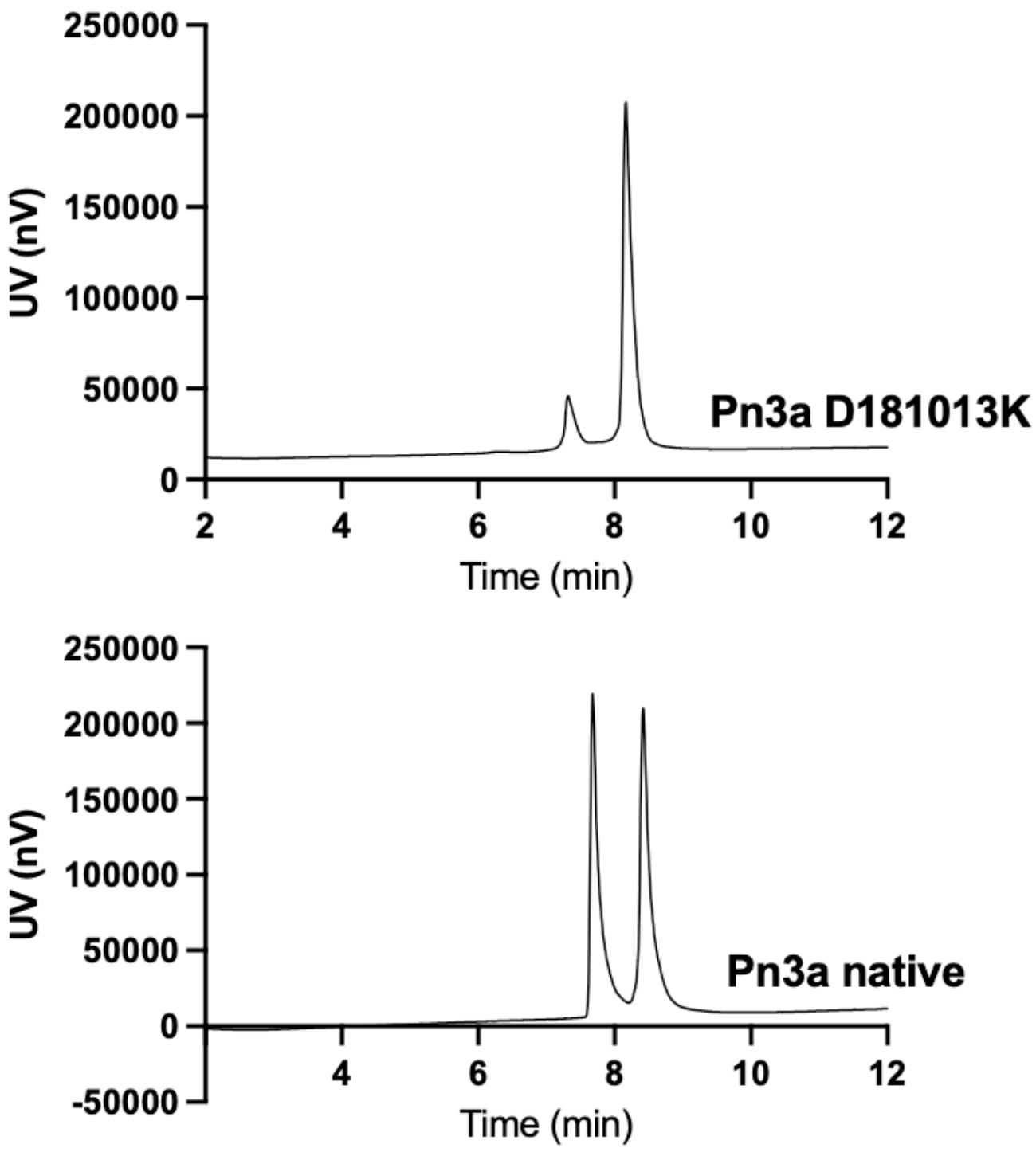

4, On page 15, line 408, the E818G mutation neutralizes the charge but also substantially reduces the side chain volume. Please discuss whether the observed shift could be partially attributed to this structural change rather than purely electrostatic effects.

We agree with the reviewer that the E818G substitution removes the negative charge while also substantially reducing side-chain volume, and therefore we cannot fully exclude a contribution from local structural changes in the absence of structures for each mutant channel. However, the direction and pattern of the functional effects argue against a simple steric explanation. Glycine has only a hydrogen atom as its side chain and is therefore markedly smaller than glutamate. If the depolarising shift produced by E818G were primarily caused by reduced side-chain bulk, one might expect this substitution to relieve steric constraints on voltage-sensor movement rather than hinder activation. Instead, E818G produces a depolarising shift in V1/2, consistent with impaired upward movement of the DII voltage sensor following loss of the native negative charge. Moreover, substitution with lysine, which retains substantial side-chain bulk and length compared with glutamate but reverses the charge, produces an even larger depolarising shift. Thus, although local structural effects cannot be completely ruled out, the concordant effects of charge neutralisation and charge reversal support the interpretation that the observed V1/2 shifts are driven predominantly by electrostatic effects rather than by side-chain volume alone.

To address this point, we have included additional discussion in the manuscript:

"In addition to altering electrostatic properties, substitutions at E818 also changed side-chain size and chemistry, raising the possibility that steric effects could contribute to the observed gating shifts. However, the functional effects are unlikely to be explained by altered side-chain volume alone. Glycine is substantially smaller than glutamate, and if steric constraints were dominant, reducing side-chain bulk would be expected to facilitate voltage-sensor movement and favour activation. Instead, E818G produced a depolarising shift and impaired activation. Conversely, E818K, which restores substantial side-chain size while reversing the charge, caused an even larger depolarising shift. Together, these findings support a model in which the effects of E818 substitutions are driven primarily by electrostatic interactions rather than steric constraints, although additional local structural contributions cannot be excluded in the absence of high-resolution mutant channel structures."

5, Please specify the voltage at which the concentration-response curves of Pn3a and its analogue were measured. Given that they differentially shift the voltage dependence of Nav1.7 activation (Fig. 4E), the potency may vary at voltages more positive than 0 mV.

The reviewer raises an important point. All concentration-response curves were determined from depolarisations to -20 mV, as detailed in the Methods. Although Pn3a and its analogue differentially shift the voltage dependence of Nav1.7 activation, the G-V curves show that channel activation is near-maximal and comparable between peptides at -20 mV. We agree that, in principle, peptide potency could be assessed across a range of voltages, or from peak current at the voltage that elicits maximal channel opening for each condition. However, this would require determination of full I-V curves at each peptide concentration, which was not feasible. We therefore used -20 mV, where shifts in activation are expected to have minimal impact on channel availability, allowing direct comparison of peptide potency under identical recording conditions.

6, Per journal guidelines, all data should be reported as mean {plus minus} SD rather than mean {plus minus} SEM. Additionally, please provide exact p-values for all comparisons.

We now report all data as mean +/- SD and report all exact p-values in the manuscript.

7, In Tables 3 and 4, please include the number of experimental cells (n) for each construct, including WT and mutants.

We have amended Table 3 and 4 to include n values.

8, In Figure 4B (below), the residues are labeled with the same names for both Pn3a and Pn3a (+++). Please update the labels to correctly distinguish between the two peptides.

We thank the reviewer for bringing this oversight to our attention and have now corrected the peptide residue labelling.

9, Please use the abbreviation "Na_V" consistently instead of "Nav" across all four figures.

We have corrected all instances to "Na_V" in the figures.

Referee #2:

This manuscript examines the structural characteristics that tune the voltage sensitivity of sodium channels. By comparing the sequence and activation thresholds of Nav1.7 and Nav1.8 they are able to pinpoint the importance of charges in the domain II voltage sensor extracellular loops, which is tested through careful mutagenesis studies. They propose an electrostatic mechanism of this effect tested through the introduction of calcium and by tuning the charge on peptides that bind to this region of the protein. Overall, this is an excellent paper. It is very clearly written and provides valuable information that improves our understanding of voltage gated channels. The experiments are appropriate and well conducted. I have a few comments and suggestions that mostly relate to the mechanistic interpretation.

It is proposed that the impact of the loop charges on the threshold for activation is via an electrostatic effect. The impact of Ca2+ and the Pn3a toxin support this. However, there is some data that is harder to rationalise. Most notably, the introduction of 2 positive residues to Nav1.7 to reproduce the Nav1.8 sequence DVEG -> KKGS has the same or greater effect as changing DVEG -> KKKK. The electrostatic interpretation should suggest that the threshold would be more depolarised with KKKK than KKGS. How is this rationalised? I feel this point needs to be more carefully considered.

The reviewer raises an important point. The most likely interpretation is that the position and exposure of the introduced charges, rather than simply the total number of charged residues in the loop, determine their effect on voltage-sensor activation. Thus, not all charged residues in this region are expected to contribute equally to the local electrostatic environment experienced by the DII voltage sensor, as also suggested by the distinct effects of individual substitutions at E818. In the DVEG→KKGS mutant, the two introduced lysine residues may occupy positions and/or side-chain orientations that are particularly effective at altering the electrostatic field near the voltage sensor. By contrast, in DVEG→KKKK, the additional lysines may be less favourably positioned, less solvent exposed, or may alter local loop conformation such that they do not produce an additive depolarising effect. Therefore, the comparable effects of KKGS and KKKK do not necessarily contradict an electrostatic mechanism, but indicate that the functional effect depends on the spatial presentation of charge rather than charge number alone. We have expanded the discussion to clarify this point.

"In $Na_V1.7$, the more pronounced depolarising shift observed with the KKGS substitution compared to KKKK suggests that activation is not governed solely by the number of

positive charges within the DII S3–S4 loop but by the spatial presentation of charged residues relative to the S4 voltage sensor. In particular, the position, orientation, and solvent exposure of individual side chains are likely critical in shaping the local electrostatic environment that stabilises distinct voltage-sensor conformations. Under this framework, the two lysines introduced in KKGS likely occupy positions that are particularly effective at modulating S4 movement, whereas additional lysines in KKKK may not contribute additively due to suboptimal orientation, limited exposure, or local conformational constraints within the loop. This interpretation is consistent with the distinct effects observed for individual substitutions at D816 and E818 and supports an electrostatic mechanism in which charge position and presentation, rather than number of charges alone, is a key determinant of functional outcome."

The effect of Ca2+ is said to be through electrostatic screening rather than specific binding. This is very likely given the data, but I was not entirely convinced that binding could be ruled out. One way to test this would be to show that increasing Na+ concentration can have the same effect as adding Ca2+ - noting that four times as much sodium would be required to have the same screening effect.

We agree with the reviewer that specific Ca^{2+} binding cannot be absolutely excluded. However, several observations favour electrostatic screening as the predominant mechanism. First, Ca^{2+} binding to positively charged loops would be electrostatically unfavourable, while binding to negatively charged loops would be expected to neutralise negative charge and therefore does not readily explain the observed effects on voltage sensitivity. Second, we have previously shown that increasing extracellular Na^{+} concentration produces a similar effect to Ca^{2+}, consistent with charge screening rather than Ca^{2+}-specific binding. We have added this point to the Discussion.

Discussion text:

"These results indicate that Ca^{2+} is unlikely to act through a specific binding site in this region but instead modulates activation by screening the local electrostatic environment sensed by the S4 voltage sensor, highlighting the strong influence of clustered loop charges on voltage sensitivity. Consistent with an electrostatic screening mechanism, we have previously shown that decreasing extracellular Na^{+} concentration produces a similar effect to decreasing extracellular Ca^{2+} (Thapa *et al.*, 2024)."

An alternative mechanistic explanation is that these mutations effectively increase the charge on the S4 helix as they are on the loop region adjacent to the top of the helix. Increased charge would yield a strong attraction to the negative interior of the cell, meaning that the cytoplasm has to depolarise further to induce S4 movement. I am not

sure this is contrary to the electrostatic proposal given in the paper as both relate to electrostatic interactions, this, however, gives it a more structural emphasis. The authors have been careful to be factual and not provide unwarranted speculation, but I am at least intrigued to consider how the electrostatic tuning works at a structural level.

We agree with the reviewer that this is a plausible mechanistic explanation and that it is not contrary to the electrostatic interpretation proposed in the manuscript. Rather, it provides a potential structural framework for how altered loop charge could influence S4 movement. Specifically, introduction of positive charge into the loop adjacent to the extracellular end of S4 may effectively increase the positive electrostatic environment associated with the voltage sensor, thereby stabilising S4 in a resting/downward position and requiring stronger depolarisation to promote outward movement. As the reviewer notes, definitive testing of this mechanism would require structural information for the mutant channels, which is currently not available. We have therefore added this possibility to the Discussion.

Discussion text:

"One possible structural interpretation of these findings is that positive charges introduced into the DII S3-S4 loop adjacent to the extracellular end of S4 may effectively increase the positive electrostatic environment associated with the voltage sensor. This could stabilise S4 in its resting/downward position, for example through enhanced electrostatic attraction toward the intracellular negative potential, thereby requiring stronger depolarisation to drive outward S4 movement. This mechanism remains speculative in the absence of structures of the mutant channels but provides a plausible structural framework for how loop charge may tune voltage-sensor activation."

Minor comments:

Line 111 : Given that Nav can contain beta subunits, I recommend changing the wording of

"NaV channels are composed of a single alpha subunit organized" to "Nav Contain a single alpha subunit ...

We have amended the wording according to the suggestion.

Figure 3B, I recommend adding a label to be clear this is DII

We have amended Fig 3B according to the reviewer's suggestion.

Line 395: I recommend softening the wording about charged pair interactions. It is possible that such interactions exist between residues not considered in this analysis.

We agree with the reviewer. We have amended the wording to better reflect that we experimentally assessed only the key paired interactions identified from existing structures, while acknowledging that additional interactions may exist that were not assessed in this study. Importantly, mutations of nearby residues predicted to form charge-pair interactions with S4 gating charges, including E753, E811 and N769, had little impact on activation. These data suggest that, for the interactions tested, voltage sensitivity is more strongly influenced by the local electrostatic potential of the S3–S4 loop than by discrete pairwise interactions with these residues.

Updated discussion

"While these mutations could, in principle, introduce or disrupt specific charge–charge interactions with S4 gating residues, substitution of neighbouring residues predicted to interact with S4 gating charges, including E753, E811 and N769, had little impact on activation. Collectively, these data do not support a dominant role for discrete pairwise interactions at these positions, but instead indicate that voltage sensitivity is primarily governed by the broader local electrostatic environment generated by the S3–S4 loop."

Line 426: It would nice to be clear about what is known structurally for the Pn3a - Nav1.7 interaction. Even a statement such as "while there is not cryo-EM structure of the Pn3a - Nav1.7 complex previous mutation studies suggest that K22 and K24 directly interact with Nav1.7 possible by binding to D816 and E818" and include the citation.

We thank the reviewer for this suggestion. To date, no cryo-EM structure of the Pn3a-Na_V1.7 complex has been reported, and current knowledge of this interaction is therefore based primarily on functional data. Previous mutagenesis studies suggest that Pn3a residues K22 and K24 contribute to NaV1.7 binding, potentially through interactions with acidic residues in the DII S3–S4 loop, including D816 and E818. We have added this clarification and the relevant citation to the Discussion.

Manuscript text:

Although no cryo-EM structure of the Pn3a-Na_V1.7 complex is currently available, previous functional and mutagenesis studies suggest that Pn3a residues K22 and K24 contribute to Na_V1.7 binding, potentially through interactions with acidic residues in the DII S3–S4 loop, including D816 and E818 (Mueller *et al.*, 2020), suggesting that Pn3a can

depolarize activation either by neutralizing these negative charges and/or by introducing additional positive charge.

Dear Dr Deuis,

Re: JP-RP-2026-291286R1 "**Electrostatic Tuning of Na_V Channel Activation by the Domain II S3-S4 Extracellular Loop** " by Ashvriya Thapa, Lotten Ragnarsson, Hue Tran, Angelo Keramidas, Jennifer R Deuis, and Irina Vetter

We are pleased to tell you that your paper has been accepted for publication in The Journal of Physiology.

The last Word (or similar) version of the manuscript provided will be used by the Production Editor to prepare your proof. When this is ready you will receive an email containing a link to Wiley's Online Proofing System. The proof should be thoroughly checked and corrected as promptly as possible.

Authors should note that it is too late at this point to offer corrections prior to proofreading. Major corrections at proof stage, such as changes to figures, will be referred to the Editors for approval before they can be incorporated. Only minor changes, such as to style and consistency, should be made at proof stage. Changes that need to be made after proof stage will usually require a formal correction notice.

All queries at proof stage should be sent to: TJP@wiley.com.

Following copyediting and typesetting the accepted version will be published online.

Yours sincerely,

Peying Fong
Senior Editor
The Journal of Physiology

IMPORTANT POINTS TO NOTE FOLLOWING ACCEPTANCE OF YOUR PAPER:

• IMPORTANT NOTICE ABOUT OPEN ACCESS: To assist authors whose funding agencies mandate immediate public access to published research findings, The Journal of Physiology allows authors to pay an Open Access (OA) fee to have their papers made freely available immediately on publication.

The Corresponding Author will receive an email from Wiley with details on how to register or log in to Wiley Authors where you will be able to place an order.

• You can check if your funder or institution has a Wiley Open Access Account here:
https://authors.wiley.com/author-resources/Journal-Authors/open-access/author-compliance-tool.html

MEMBER DISCOUNT: Corresponding authors who are Members of the Physiological Society are eligible for a 20% discount on the cost of the Article Processing Charge (APC). To claim this discount, the Corresponding Author will need to log in to the Physiological Society Membership portal and retrieve the discount code (https://www.physoc.org/journals-and-media/journals/journal-discount-for-members/). This code should then be quoted in the billing section of Wiley Authors when selecting the open access option.

• TRANSPARENT PEER REVIEW POLICY: To improve the transparency of its peer review process, The Journal of Physiology publishes online as supporting information the peer review history of all articles accepted for publication. Readers will have access to decision letters, including Editors' comments and referee reports, for each version of the manuscript as well as any author responses to peer review comments. Referees can decide whether or not they wish to be named on the peer review history document.

• You can help your research get the attention it deserves! Check out Wiley's free Promotion Guide for best-practice recommendations for promoting your work at: www.wileyauthors.com/eeo/guide. You can learn more about Wiley Editing Services which offers professional video, design, and writing services to create shareable video abstracts, infographics, conference posters, lay summaries, and research news stories for your research at: www.wileyauthors.com/eeo/promotion.

• If you would like to receive our 'Research Roundup', a monthly newsletter highlighting the cutting-edge research published in The Physiological Society's family of journals (The Journal of Physiology, Experimental Physiology, Physiological Reports, The Journal of Nutritional Physiology and The Journal of Precision Medicine: Health and Disease), please click this link, fill in your name and email address and select 'Research Roundup':
https://www.physoc.org/journals-and-media/membernews

EDITOR COMMENTS

Reviewing Editor:

The authors have done a nice job addressing all of the concerns and comments raised by the reviewers. No additional concerns are noted.

Senior Editor:

Thank you for addressing all remaining comments. Both Expert Referees and the Reviewing Editor agree that you've done a superb job in doing so. At this time, I am happy to let you know your manuscript is ready for final acceptance. On behalf of The Journal of Physiology, please accept thanks for contributing this fine study.

REFEREE COMMENTS

Referee #1:

All of my questions have been addressed, and I have no further comments.

Referee #2:

The authors have done an excellent job revising the manuscript addressing all my queries and concerns.
